# Single-Cell Atlas of Lineage States, Tumor Microenvironment, and Subtype-Specific Expression Programs in Gastric Cancer

**DOI:** 10.1158/2159-8290.CD-21-0683

**Published:** 2022-03-08

**Authors:** Vikrant Kumar, Kalpana Ramnarayanan, Raghav Sundar, Nisha Padmanabhan, Supriya Srivastava, Mayu Koiwa, Tadahito Yasuda, Vivien Koh, Kie Kyon Huang, Su Ting Tay, Shamaine Wei Ting Ho, Angie Lay Keng Tan, Takatsugu Ishimoto, Guowei Kim, Asim Shabbir, Qingfeng Chen, Biyan Zhang, Shengli Xu, Kong-Peng Lam, Huey Yew Jeffrey Lum, Ming Teh, Wei Peng Yong, Jimmy Bok Yan So, Patrick Tan

**Affiliations:** 1 Cancer and Stem Cell Biology Program, Duke-NUS Medical School, Singapore.; 2 Department of Haematology-Oncology, National University Cancer Institute, National University Health System, Singapore.; 3 Yong Loo Lin School of Medicine, National University of Singapore, Singapore.; 4 The N.1 Institute for Health, National University of Singapore, Singapore.; 5 Singapore Gastric Cancer Consortium, Singapore.; 6 Department of Medicine, National University of Singapore, Singapore.; 7 Gastrointestinal Cancer Biology, International Research Center for Medical Sciences (IRCMS), Kumamoto University, Kumamoto, Japan.; 8 Cancer Science Institute of Singapore, National University of Singapore, Singapore.; 9 Department of Surgery, University Surgical Cluster, National University Health System, Singapore.; 10 Institute of Molecular and Cell Biology, Agency for Science, Technology and Research, Singapore.; 11 Department of Microbiology and Immunology, Yong Loo Lin School of Medicine, Singapore.; 12 Singapore Immunology Network (SIgN), A*STAR, Singapore.; 13 Department of Physiology, National University of Singapore, Singapore.; 14 School of Biological Sciences, Nanyang Technological University, Singapore.; 15 Department of Pathology, National University Health System, Singapore.; 16 Division of Surgical Oncology, National University Cancer Institute, Singapore.; 17 Genome Institute of Singapore, Agency for Science, Technology and Research, Singapore.; 18 SingHealth/Duke-NUS Institute of Precision Medicine, National Heart Centre Singapore, Singapore.

## Abstract

Gastric cancer heterogeneity represents a barrier to disease management. We generated a comprehensive single-cell atlas of gastric cancer (>200,000 cells) comprising 48 samples from 31 patients across clinical stages and histologic subtypes. We identified 34 distinct cell-lineage states including novel rare cell populations. Many lineage states exhibited distinct cancer-associated expression profiles, individually contributing to a combined tumor-wide molecular collage. We observed increased plasma cell proportions in diffuse-type tumors associated with epithelial-resident *KLF2* and stage-wise accrual of cancer-associated fibroblast subpopulations marked by high *INHBA* and *FAP* coexpression. Single-cell comparisons between patient-derived organoids (PDO) and primary tumors highlighted inter- and intralineage similarities and differences, demarcating molecular boundaries of PDOs as experimental models. We complemented these findings by spatial transcriptomics, orthogonal validation in independent bulk RNA-sequencing cohorts, and functional demonstration using *in vitro* and *in vivo* models. Our results provide a high-resolution molecular resource of intra- and interpatient lineage states across distinct gastric cancer subtypes.

**Significance:**

We profiled gastric malignancies at single-cell resolution and identified increased plasma cell proportions as a novel feature of diffuse-type tumors. We also uncovered distinct cancer-associated fibroblast subtypes with *INHBA–FAP*-high cell populations as predictors of poor clinical prognosis. Our findings highlight potential origins of deregulated cell states in the gastric tumor ecosystem.

*This article is highlighted in the In This Issue feature, p. 587*

## Teaser

Single-cell RNA sequencing analysis of over 200,000 gastric cancer cells forms a comprehensive molecular atlas, identifying novel cell lineages and unique interactions between tumor cells and the surrounding microenvironment.

## Introduction

Gastric cancer is a leading cause of global cancer morbidity and mortality (1) with particularly high incidence in Asia, Eastern Europe, and Central America (2). Between individual patients, gastric tumors frequently exhibit high levels of histologic, transcriptomic, and (epi)genomic variation, with distinct clinical behaviors and treatment response (“interpatient heterogeneity”). Factoring this heterogeneity into gastric cancer clinical management, and identifying molecular pathways driving hallmarks of gastric cancer variation, represent important goals for improving patient outcomes. Although progress has been made in defining specific molecular subtypes of gastric cancer through consortia such as The Cancer Genome Atlas (TCGA) and Asian Cancer Research Group (ACRG; refs. 3, 4), tangible improvements in patient outcomes based on these findings have been modest, compounded by the growing recognition that gastric cancers also exhibit high levels of within-patient heterogeneity [“intrapatient heterogeneity” (ITH)]. High-resolution studies probing the molecular extent of gastric cancer ITH across a wide range of patients with gastric cancer are thus required (5) to understand key principles governing gastric cancer evolution, selection, and adaptation and how clinical care pathways should be adapted to manage gastric cancer ITH.

Using “bulk-transcriptome” experimental methods, we and others have previously established that each gastric tumor possesses a personalized expression profile comprising distinct transcriptional programs, contributed by both cancer epithelial cells and other cell types in the tumor microenvironment (TME; refs. 6, 7). However, our understanding of mechanisms by which TME-resident cell types such as immune cells, fibroblasts, and blood vessels drive gastric cancer phenotypes and clinical trajectories remains nascent (8). Although advanced bioinformatic programs have been designed to decompose bulk sequencing data into lineage-specific constituent programs, these deconvolution algorithms are often not able to discern fine-scale tissue lineages, relationships between lineages, rare cell populations, and cell–cell interactions (9). To tackle these challenges, single-cell RNA sequencing (scRNA-seq) is proving to be a powerful tool for characterizing gene expression across thousands of cells simultaneously, enabling comprehensive profiling of different cell types in tumors in distinct biological states and conditions (10). In gastric cancer, recent scRNA-seq studies have provided unique insights on different aspects of gastric tumor biology. For example, Wang and colleagues performed scRNA-seq on malignant cells from ascitic fluids of patients with gastric cancer to develop a prognostic signature based on features of malignant peritoneal cells (11). Other scRNA-seq studies of primary gastric cancer tumors have been performed, but on limited numbers of samples and cells (8–13 patients, 27,000 to 55,000 cells per study; refs. 12–14). Another limitation of current single-cell sequencing platforms is the requirement for tissue dissociation, which leads to loss of spatial information. To address this, newer “spatial transcriptomic” platforms such as digital spatial profiling (DSP), *in situ* sequencing, and MERFISH have been developed that retain spatial architecture, thereby allowing analysis of tumor–TME interactions at unprecedented depth (15–17).

Here, we performed scRNA-seq on an expanded cohort of more than 30 patients with gastric cancer with different subtypes and stages, across a large number of cells (>200,000 cells), to generate a comprehensive singe-cell landscape of gastric cancer encompassing both inter- and intratumoral heterogeneity. We discovered unique and novel features of the gastric TME, including an increased proportion of plasma cells in diffuse-type tumors, and a role for *INHBA*, a subunit of activin–inhibin complexes, in specific subtypes of cancer-associated fibroblasts (CAF). We also performed spatial transcriptomic analysis on these samples to geographically validate predicted intercell and intracell relationships *in situ*, and further reinforced our findings using functional assays and conventional IHC.

## Results

### scRNA-seq of ~200,000 Gastric Cancer Cells Identifies Diverse Tissue-Lineage States, Cell Fate Trajectories, and Rare Cell Populations

**Figure 1.**
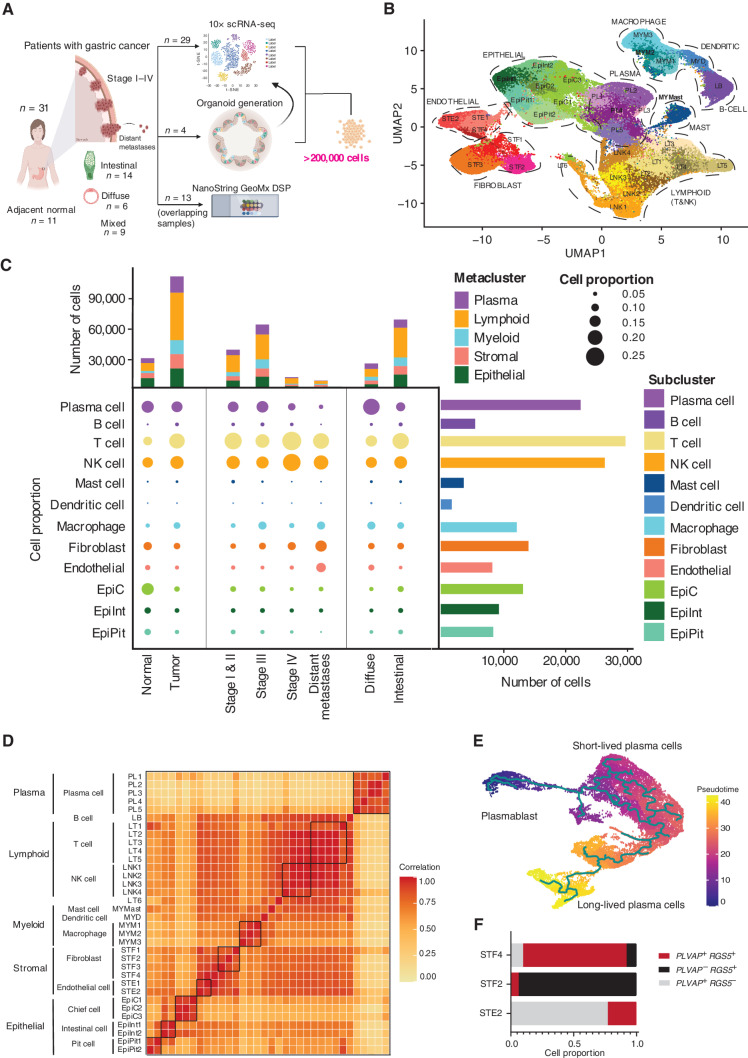
scRNA-seq of gastric tumor and normal samples defines 34 cell states including rare cell populations. **A,** Schematic representation of experimental design and techniques used in this study. Thirty-one unique patients with gastric cancer undergoing surgical resection or endoscopy had tumor samples (*n* = 31) and adjacent normal samples (*n* = 11) harvested for analysis. Tumors ranged from stage I to IV and included samples of both primary tumors, distant (peritoneal) metastases, and matched normal gastric tissues. Twenty-nine tumors had scRNA-seq performed using the 10× platform (along with 11 adjacent normal tissues). Four patients had PDOs generated from their tumors (4 tumors + 4 adjacent normal), which were also sequenced by 10× scRNA-seq. A subset of 13 samples also had DSP performed using the NanoString GeoMx platform (10 tumor + 3 normal). In total, more than 200,000 cells were sequenced in this study. **B,** Uniform Manifold Approximation and Projection (UMAP) of 152,423 cells representing 34 unique cell states color-coded by their corresponding cell lineage or subtype. Each dot in the UMAP represents a single cell. **C,** Cell-lineage compositions of gastric cancer and normal samples inferred by scRNA-seq data. Middle (bubble plot), cell subclusters (rows) by tumor versus normal, stage, and gastric cancer histologic subtype (diffuse vs. intestinal). The size of the circle represents the cell proportion of each specific cell lineage/type. The circles are color-coded by defined cell lineages/types as shown in **B**. The stacked bar graph on the top shows the number of cells in each meta-cluster for each category. The histogram on the right shows the absolute cell numbers in each subcluster. **D,** Cluster–cluster heat map of gene-expression data of all 34 cell states across all samples using Pearson correlation matrix. Darker colors correspond to higher correlation. **E,** Pseudotime analysis of plasma metacluster generated using Monocle. The trajectory was rooted against the plasmablasts. Pseudotime analysis demonstrates different stages of plasma cell differentiation and maturation including plasmablasts, short-lived plasma cells, and long-lived plasma cells. **F,** Expression of *PLVAP* and *RGS5* in endothelial (STE2) and fibroblast (STF2 and STF4) clusters. Doublets were identified and filtered out using DoubletFinder. *PLVAP*^+^*RGS5*^−^ cells are predominantly present in the endothelial cluster (STE2). *PLVAP*^−^*RGS5*^+^ cells are predominant in the fibroblast cluster (STF2). The STF4 cluster shows cells expressing *PLVAP*^+^*RGS5*^+^, suggestive of a rare mixed-lineage population.

Droplet-based scRNA-seq (10× Genomics) was performed on 48 surgical resection and biopsy samples across 31 unique individuals with gastric cancer, ranging from stages I to IV, distinct histologic subtypes (diffuse and intestinal), molecular subtypes (TCGA), primary tumors to distant (peritoneal) metastases, and matched normal gastric tissues (Fig. 1A; Supplementary Table S1). We also performed scRNA-seq on tumor and matched normal gastric cancer patient-derived organoids (PDO) to investigate the effects of *in vitro* culture on cancer-intrinsic and TME signatures (Fig. 1A; Supplementary Table S1). After quality control (QC) and removal of batch effects (18), 200,954 single cells were included in the final data set. For 13 samples [10 tumor and 3 normal; 156 regions of interest (ROI)], we also performed spatial transcriptomics using the DSP platform (NanoString GeoMx) to gain insights into *in situ* geographic and spatial relationships linking discrete cell states and molecular interactions.

We first performed dimensionality reduction on 152,423 cells derived from 40 primary samples (29 tumor and 11 normal, of which 10 were matched). This analysis revealed 34 unique tissue states (Fig. 1B). Using tissue type–specific canonical markers defined in the literature (ref. 14; Supplementary Fig. S1), the cell states were broadly categorized into five major cell types, referred to as “metaclusters” (myeloid, lymphoid, plasma, epithelial, and stromal; Fig. 1C; Supplementary Fig. S2A). Supporting the supervised cell type–specific marker analysis, an unsupervised global clustering similarity matrix also classified the cell states into five metaclusters (Fig. 1D). Alternative clustering algorithms (e.g., integrative nonnegative matrix factorization) confirmed the molecular distinctiveness of each cluster, and comparison of global versus metacluster-specific clustering approaches revealed similar results (Supplementary Fig. S2B–S2E).

Overall, immune-cell populations dominated the cell states (21 of 34 states). These included: (i) a “myeloid metacluster” (five cell states), consisting of a mast cell cluster (*KIT* positive: “MAST”), a dendritic cell cluster (*PLD4* positive: “MYD”), and three macrophage clusters (*CD163* positive: “MYM1–3”); (ii) a “lymphoid metacluster” (11 cell states), consisting of six T-cell clusters, mapping to effector and naïve CD8 T cells (*CD8A*, *GZMH, GZMM*, and *NKG7*; LT1–2), naïve and helper CD4 T cells (*CCR7* and *STAT4;* LT3–4), regulatory T cells (Treg; *IL2RA* and *STAT3*; LT5), and proliferative T cells (expressing cell-cycle genes; LT6), four natural killer (NK) cell clusters (*KLRD1* positive; LNK1–4), and one B-cell cluster (*MS4A1* positive; LB); and (iii) a “plasma metacluster” (five cell states), consisting of five mature B cells/plasma cell clusters (*TNFRSF17* positive; PL1–5; Supplementary Fig. S1; Supplementary Table S2).

The “epithelial” metacluster (seven cell states; *CDH1* positive) contained three distinct sublineages: “EpiPit,” expressing markers of mucous pit cells (*MUC5AC* and *TFF1*; EpiPit1&2); “EpiC,” expressing markers of chief cells (*LIPF* and *PGA3*; EpiC1–3); and “EpiInt,” expressing intestinal-type markers (*REG4* and *TFF3*; EpiInt1&2; Supplementary Fig. S1). EpiInt cells exhibited features of intestinal metaplasia (IM), a premalignant condition recognized histologically by gastric epithelial cells acquiring intestinal cell–type features (12). This was supported by the expression of *CDX1* and *CDX2* master transcriptional regulators, largely in EpiInt cells, which play a key role in the *trans*-differentiation of gastric epithelial cells to intestinal cell type (Supplementary Fig. S2F; ref. 19). The “stromal metacluster” (six cell states; *FN1* positive) consisted of pericytes (STF2; defined by *RGS5 and NOTCH3*), fibroblasts (STF1 and STF3; defined by *LUM* and *DCN*), and *PLVAP*-positive endothelial cell subclusters. The latter could be further partitioned into *ACKR1*-positive venular endothelial cells (STE1) or nonvenular endothelial cells (STE2; ref. 20; Supplementary Fig. S1). The remaining cluster (STF4) could not be assigned to a single cell type and is described further in the next paragraph. The cell types varied in their relative proportions across the ∼150K cells (Fig. 1C; Supplementary Fig. S2G). Specifically, lymphoid cells exhibited the largest proportion (39.7%; range, – 19.1% for T cells to 3.6% for B cells) followed by epithelial cells (20%; range, 8.5% for EpiC to 5.4% for EpiPit cells). Myeloid, stromal, and plasma cells constituted proportions of 11.4%, 14.4%, and 14.5%, respectively.

The large number of cells profiled in our study enabled us to relate distinct cell states to one another across biological state transitions. For example, single-cell trajectory and pseudotime analysis of the plasma cell metacluster demonstrated different stages of plasma cell differentiation and maturation, consistent with the known literature (ref. 21; bootstrapping *P* < 0.05 on major branching nodes; Fig. 1E). These included plasmablasts (high *XBP1* and low *SDC1*), short-lived plasma cells (high *SDC1*), and long-lived plasma cells (high *SDC1*, *STAT3*, and *IKZF3*; refs. 22–25). Short-lived plasma cells predominated the trajectory plot, whereas long-lived plasma cells exhibited relatively higher expression of *IGHA1* (IgA), consistent with increased levels of IgA detected in gastric lamina propria (ref. 26; Supplementary Fig. S2H). Similarly, single-cell trajectory analysis of macrophages revealed two distinct cell states: proinflammatory “M1-like” (high *CD163* and *S100A12*) and “M2-like” tumor-associated macrophages (high *CD163* and *FOLR2*; refs. 27 and 28; Supplementary Fig. S2I). The large number of analyzed cells also enabled characterization of a novel and rare cell type (STF4) within the stromal metacluster (0.4% of all cells; *n* = 821), expressing markers associated with both endothelial (*PLVAP*) and fibroblast (*RGS5*) lineages (Fig. 1F), possibly highlighting cells undergoing endothelial–mesenchymal transition (EndoMT), a process where endothelial cells acquire a mesenchymal or myofibroblastic phenotype. We ruled out the possibility that the double-lineage nature of STF4 cells is caused by potential technical artifacts such as doublet effects (29) and orthogonally validated the existence of the *PLVAP/RGS5* double-positive population on formalin-fixed paraffin-embedded (FFPE) sections using dual-color RNAScope (Supplementary Fig. S2J). Taken together, these results showcase the ability of scRNA-seq data, when appropriately powered, to reveal cells exhibiting diverse tissue-lineage states, different state transitions, and rare populations associated with mixed or dual lineages.

### scRNA-seq Discerns Cell State–Specific Transcriptional Patterns Associated with Gastric Cancer

**Figure 2.**
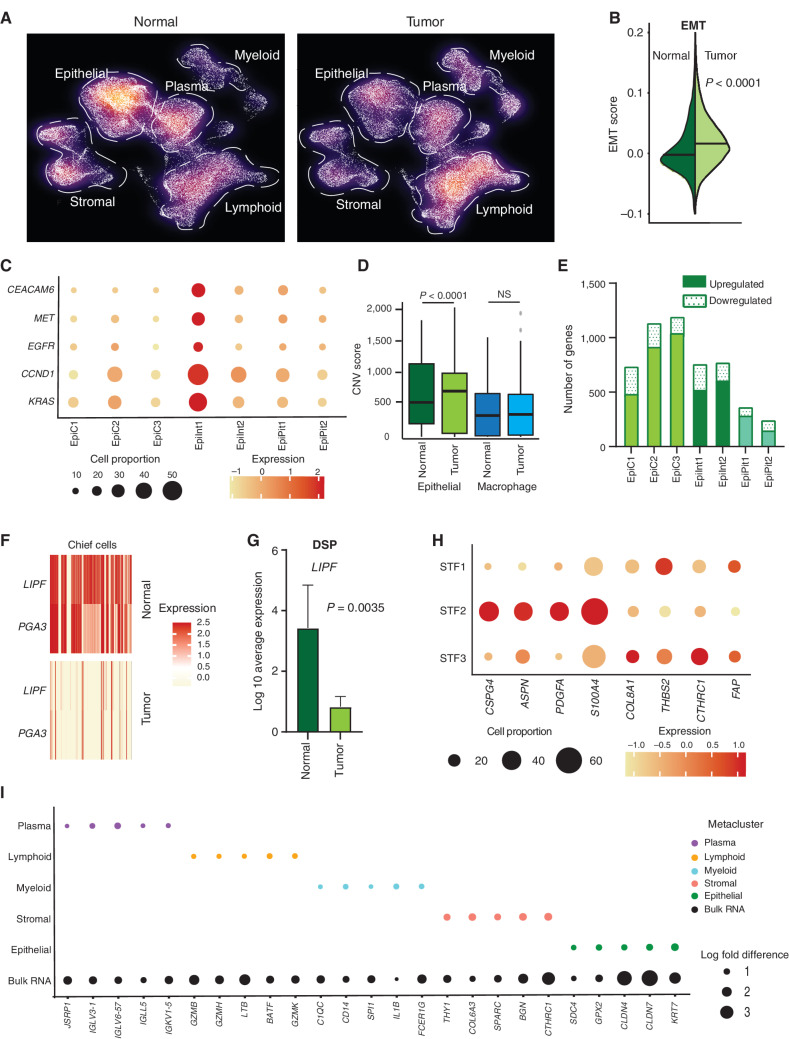
scRNA-seq deconvolutes gastric tumor programs associated with distinct cell states. **A,** Density plot of UMAP representation comparing normal and gastric tumor samples after random downsampling to approximately 30,000 cells each to allow statistical equivalence. Each dot represents a single cell. Dashed lines highlight higher proportions of epithelial cells in normal samples and myeloid cells in tumor samples. **B,** Split violin plot of EMT oncogenic gene signature score in normal and tumor cells, showing a significantly higher score in tumor cells. **C,** Bubble plot depicting the expression of gastric cancer oncogenes in tumor epithelial cell clusters. The size of the circle represents the percentage of cells expressing the gene in that specific epithelial cell cluster, whereas the color represents the average expression of the gene. **D,** Box plot depicting CNV scores for epithelial cells (green) and macrophage cells (blue) in normal and tumor samples. CNV scores were computed using InferCNV. *P* values were computed using Wilcoxon rank-sum test. **E,** Bar graph depicting differences in transcriptomic profiles between tumor and normal tissue by number of upregulated and downregulated genes in epithelial cell clusters. **F,** Heat map of *LIPF* and *PGA3* gene expression (classic chief cell marker genes) in tumor versus normal samples. Darker color signifies higher expression. **G,** Gene expression of *LIPF* by DSP analysis, in tumor epithelial cells (Pan-CK^+^) compared with normal samples (*n* = 13). **H,** Bubble plot depicting sublineage-specific expression of CAF marker genes *FAP*, *CSPG4*, *PDGFA*, *ASPN*, *S100A4*, *COL8A1*, *THBS2*, and *CTHRC1* in fibroblast clusters. STF1 and STF3 are *LUM*-associated fibroblasts, whereas STF2 comprises proangiogenic pericytes. The size of the circle represents the percentage of cells expressing the gene in that specific fibroblast cell cluster, whereas the color represents the average expression of the gene. **I,** Bubble plot depicting significant log fold differences in expression of genes between tumor and normal by metacluster mapped against the bulk RNA-seq data (five genes per metacluster are shown). The size of the circle represents the log fold change in the expression of specific genes.

To examine tumor-associated expression programs at the single-cell level, we compared primary gastric tumor samples to normal tissues (*n* = 26 tumor; *n* = 10 normal). After random downsampling to ensure statistical equivalency (Fig. 2A), the proportion of epithelial cells in tumors was lower compared with normal tissues (*P* = 0.002), whereas myeloid cells in tumors were higher (*P* = 0.05; Supplementary Table S3). The proportions of lymphoid, plasma, and stromal cells were not statistically different between tumor and normal samples.

Compared with their cognate cell states in normal tissues, tumor-associated epithelial cells exhibited higher expression of multiple oncogenic gene signatures related to epithelial–mesenchymal transition (EMT), cell motility, and cancer signatures derived from previous gastric cancer scRNA-seq studies (refs. 12–14; Fig. 2B; Supplementary Fig. S3A and S3B; Supplementary Table S4; *P* < 0.0001). When we assessed the expression status of individual gastric cancer oncogenes (e.g., *CEACAM6*, *EGFR*, *MET*, *CCND1*, and *KRAS*) among the tumor epithelial clusters, the EpiInt1 tumor epithelial cluster exhibited the largest increase (Fig. 2C). We orthogonally validated the oncogenic gene signature associations in multiple ways. First, we confirmed a significant correlation between the proportions of inferred scRNA-seq tumor cells and EpiInt1 tumor cellularity proportions measured by hematoxylin and eosin (H&E) staining (*R* = 0.64, *P* < 0.0074). Second, inferring copy-number variations (CNV) from the scRNA-seq data using approaches employed in other cancer single-cell studies (30–32), we confirmed that tumor-associated epithelial cells exhibited significantly higher numbers of inferred aberrant CNVs compared with normal epithelial cells (*P* < 0.0001). In contrast, CNV differences were not observed between macrophage populations in tumor and normal samples (Fig. 2D). Third, further supporting a substantial proportion of epithelial cells in the tumor samples as malignant, CNV profiles inferred from scRNA-seq exhibited significant concordance with CNV profiles of the same tumor samples determined by bulk whole-exome sequencing (WES; *P*-value range, 0.022 to <0.0001; Supplementary Fig. S3C). At the subcluster level, similar to their high expression of oncogenic gene signatures, EpiInt1 cells also harbored the highest proportion of CNVs (Supplementary Fig. S3D).

Comparisons in a cell state–specific manner revealed that “chief cells” and “intestinal-type cells” showed the greatest differences in transcriptomic profiles between gastric cancers and their cognate cell state in normal gastric tissues (Fig. 2E; “chief cells”: 1,232 genes/EpiC3 cluster; “intestinal-type cells”: 775 genes/EpiInt2 cluster). Pathway analysis indicated that each tumor epithelial cluster shared both common upregulated modules relevant to cancer, but also cluster-specific tumor-associated modules. For example, all tumor epithelial clusters exhibited upregulation of immune-related pathways such as “antigen presentation: folding, assembly, and peptide loading of class I MHC.” However, only EpiC3 exhibited upregulation of “MHC class II antigen presentation” and downregulation of “citric acid (TCA) cycle and respiratory electron transport” (Supplementary Table S5). Among downregulated genes, the classic chief cell markers *LIPF* and *PGA3* were among the top downregulated genes in tumor epithelial cells (Fig. 2F; *P* < 0.0001). Using spatial DSP, we confirmed the loss of *LIPF* in tumor epithelial cells (Pan-CK^+^) compared with normal samples (*n* = 13, *P* = 0.0035; Fig. 2G). Interestingly, we and others have previously identified *LIPF* as a lineage-specific target of recurrent insertion–deletion mutations in gastric cancer (33, 34).

Among stromal metacluster cell states, absolute proportions of fibroblasts (clusters STF1–4) and endothelial cells (clusters STE1 and STE2) were not significantly altered between tumor and normal samples (Fig. 2A; Supplementary Fig. S3E; Supplementary Table S3). However, in the endothelial lineage, pathway analysis revealed a conserved expression response related to extracellular matrix (ECM) remodeling between tumor and normal samples (Supplementary Table S6). Similarly, in the fibroblast lineage, two fibroblast sublineages comprising *LUM*-associated fibroblasts (STF1 and STF3) exhibited upregulation of CAF genes such as *FAP*, *COL8A1*, *THBS2*, and *CTHRC1*, whereas a third sublineage comprising proangiogenic pericytes (STF2) displayed upregulation of another set of CAF marker genes such as *CSPG4*, *PDGFA, ASPN*, and *S100A4* (Fig. 2H; Supplementary Fig. S3F). Pathway analysis indicated several common (e.g., “ECM proteoglycans”) and specific pathways individualized to each cluster (Supplementary Table S7). For example, STF2 showed upregulation of “signaling by PDGF,” whereas STF3 had upregulation of “activation of matrix metalloproteinases.”

We also investigated T-cell proportions and immune checkpoints of therapeutic interest between tumor and normal samples (35). Consistent with the prior literature (36), we observed a higher proportion of Tregs (*P* = 0.0048) and naïve CD4 T cells (*P* = 0.048) in tumors compared with normal (Supplementary Fig. S4A and S4B). T-cell receptor (TCR) sequencing revealed TCR diversity ranges of 34.3 to 994, similar to studies in other tumor types (refs. 37, 38; see Supplementary Fig. S4C for further details). Taken collectively, these results suggest that in the gastric cancer tumor ecosystem, distinct cell states from both the epithelial and tumor microenvironment likely express different oncogenic transcriptomic features—each required for different cancer hallmarks and ultimately converging and intermixing to elicit a composite tumor molecular portrait (Fig. 2I; Supplementary Fig. S4D).

### scRNA-seq Reveals a *KLF2*-Associated Plasma Cell Program in Diffuse-type Gastric Cancer

**Figure 3.**
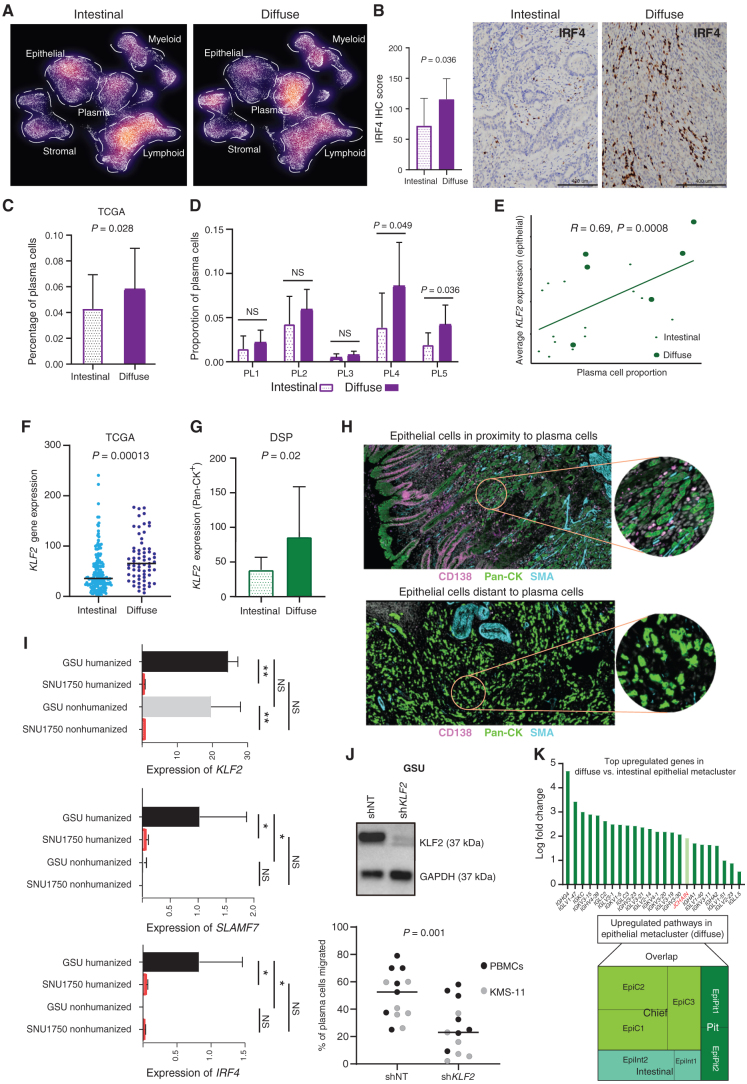
Differential TME analysis between histologic subtypes identifies increased plasma cells in diffuse-type tumors. **A,** Density plot of UMAP representation comparing diffuse and intestinal gastric cancer samples after random downsampling to approximately 25,000 cells each. Each dot represents a single cell. Dashed lines highlight a higher proportion of plasma cells in diffuse gastric cancer. **B,** IHC staining of IRF4 expression in diffuse (*n* = 5) and intestinal (*n* = 12) gastric cancer samples (scale bar, 400 μm). Bar graph showing significantly higher average IRF4 IHC score in diffuse compared with intestinal gastric cancer. **C,** Plasma cell proportions deconvoluted by CIBERSORTX in diffuse and intestinal gastric cancer in the TCGA data set (diffuse *n* = 63; intestinal *n* = 167). **D,** Bar graph showing enrichment of plasma cell proportions in PL4 and PL5 plasma subclusters in diffuse versus intestinal gastric cancer single-cell samples. **E,** Pearson correlation plot showing significant positive correlation of plasma cell proportion to average *KLF2* expression in the epithelial metacluster. **F,** Bee swarm plot showing increased *KLF2* expression in diffuse versus intestinal gastric cancer samples in the bulk RNA-seq TCGA-STAD data set. **G,** Bar graph showing increased *KLF2* expression in Pan-CK^+^ epithelial morphologic regions of diffuse versus intestinal gastric cancer by DSP (*n* = 10). **H,** DSP analysis depicting epithelial ROIs proximal (top) and distal to plasma cells (bottom). Analysis is based on immunofluorescence staining for Pan-CK (epithelial, green), CD138 (plasma, pink), smooth muscle actin (SMA; fibroblast, cyan), and DAPI (blue). Each circular ROI is 300 μm in diameter. **I,** Bar graph showing expression of *KLF2*, *IRF4*, and *SLAMF7* genes in GSU humanized and nonhumanized mice against SNU1750 humanized and nonhumanized mice. *, *P* < 0.05; **, *P* < 0.01. **J,** Western blot (top) showing stable knockdown of *KLF2* in gastric cancer cell line GSU (sh*KLF2*) compared with shNT (nontargeting) control. Loss of *KLF2* in GSU significantly reduces migration of plasma cells derived from peripheral blood mononuclear cells (PBMC) and multiple myeloma cell line KMS-11 (*N* = 17; bottom). **K,** Upregulation of immunoglobulin genes in diffuse versus intestinal epithelial metacluster (top). Tree map shows the overlap of upregulated pathways in epithelial metacluster versus subclusters, with the EpiC cluster showing the greatest overlap (bottom).

Lauren's gastric cancer classification has genomic, clinical, and prognostic value (39). We analyzed our scRNA-seq data set comparing diffuse-type (*n* = 6) and intestinal-type (*n* = 14) tumors, after random downsampling to achieve subtype matching. The relative proportion of plasma cells was higher in diffuse compared with intestinal gastric cancers (*P* = 0.05). In contrast, epithelial cells were lower in diffuse gastric cancers (*P* = 0.04), with intestinal gastric cancers exhibiting higher proportions of epithelial subclusters and inferred CNVs (*P* = 0.029; Supplementary Fig. S5A). There were no statistically significant differences in the lymphoid, myeloid, and stromal cell populations (Supplementary Table S8; Fig. 3A). We confirmed an increased level of plasma cells in diffuse versus intestinal tumors using IRF4 (a plasma cell marker) IHC on a subset of tumors (*n* = 17, *P* = 0.036; Fig. 3B; Supplementary Fig. S5B). We also independently verified the increased level of plasma cells in diffuse-type gastric cancers by reanalyzing bulk RNA-seq gastric cancer TCGA data using CIBERSORTx deconvolution (*P* = 0.028; Fig. 3C). Performing sublineage analysis in single-cell data, we established that the enrichment of plasma cells in diffuse-type gastric cancer was primarily driven by plasma cell clusters PL4 and PL5 (*P* < 0.05; Fig. 3D). Pathway analysis comparing PL4–5 against PL1–3 in tumor samples indicated upregulation of cytokine and interleukin signaling and downregulation of CD22-mediated B-cell receptor (BCR) regulation in PL4–5 (Supplementary Table S9).

*KLF2* is a gene previously shown to regulate homing of plasma cells in multiple myeloma (40, 41). We hypothesized that the increased recruitment of plasma cells in diffuse-type gastric cancers might be mediated by *KLF2* activity. To test this hypothesis, we compared *KLF2* expression between diffuse- and intestinal-subtype gastric cancers across the various cell states. Compared with intestinal-type cell states, plasma cells and epithelial cell clusters in diffuse-type gastric cancers expressed increased *KLF2* expression in >50% of cells with log-fold difference >0.5 (Supplementary Fig. S5C). Notably, we observed significant correlations only between plasma cell proportions and *KLF2*-expressing epithelial cells (*R* = 0.69, *P* = 0.0008) and not between plasma cell proportions and *KLF2*-expressing plasma cells (*R* = 0.35, *P* = 0.13; Fig. 3E; Supplementary Fig. S5D). This finding suggests that high-*KLF2*–expressing epithelial cells may be associated with plasma cell recruitment in diffuse-type gastric cancer. Sublineage analysis of diffuse-type epithelial cells revealed the highest *KLF2* expression in EpiC clusters (log fold from 1.7 to 0.8, compared with EpiC intestinal-type cells; Supplementary Table S10). To further confirm *KLF2* expression differences between intestinal-type and diffuse-type gastric cancers, we analyzed epigenomic promoter activity [as measured by H3K27ac chromatin immunoprecipitation sequencing (ChIP-seq)] and gene expression of *KLF2* in an independent bulk RNA data set of 24 gastric cancers (9 diffuse-type and 14 intestinal-type). Both *KLF2* H3K27ac promoter levels and gene expression were higher in diffuse compared with intestinal gastric cancers (*P* < 0.05), with good correlations between promoter signals and gene expression (*R* = 0.55, *P* = 0.005; Supplementary Fig. S5E). In a second independent bulk RNA-seq data set (gastric cancer TCGA), we further confirmed increased *KLF2* expression in diffuse-type compared with intestinal-type tumors (*P* = 0.00013), with boundaries of variance similar to with other credentialed oncogenes such as *ERBB2* and *HNF4α* associated with intestinal-type gastric cancer (Fig. 3F; Supplementary Fig. S5F; refs. 42, 43). To explore *KLF2* differences in a spatial context, we then used DSP analysis to study the expression of *KLF2* in epithelial cells (Pan-CK^+^) and confirmed higher *KLF2* transcript levels in diffuse-type epithelial cells compared with intestinal-type epithelial cells (*P* = 0.02; *n* = 10; Fig. 3G). Using the same platform, we next studied *KLF2* expression in epithelial cells (Pan-CK^+^) in the context of proximity to plasma cells (CD138^+^; Epi^prox^ vs. Epi^dist^). We observed a trend toward higher *KLF2* expression in epithelial cells in close proximity to plasma cells compared with distal epithelial cells (*P* = 0.096; Fig. 3H; Supplementary Fig. S5G).

To explore dynamic temporal interactions between *KLF2* expression in tumor epithelial cells with host plasma cells, we used a humanized mouse cancer *in vivo* model. Immune-deficient mouse pups were engrafted with human umbilical cord blood CD34^+^ cells, and mice with postengraftment human immune-cell reconstitution (termed “humanized mice”) were selected for the study. We investigated *KLF2*^pos^ (GSU) and *KLF2*^neg^ (SNU1750) diffuse-type gastric cancer tumors grown in humanized mice compared with immune-deficient mice (Fig. 3I). In agreement with our results in primary gastric cancers, *KLF2*^pos^ xenografts had high expression of plasma cell markers *IRF4* and *SLAMF7* in the humanized mice only, reflecting increased plasma cell recruitment associated with *KLF2*. To further demonstrate a causative role for epithelial *KLF2* in plasma cell recruitment, we then performed *in vitro* migration assays using plasma cells derived from peripheral blood mononuclear cells (PBMC) or KMS-11 (a multiple myeloma cell line) cocultured with *KLF2*-positive gastric cancer cells. Knockdown of *KLF2* expression in two independent *KLF2*-positive gastric cancer cell lines (GSU and LMSU) was sufficient to significantly decrease plasma cell migration (*P* = 0.001; Fig. 3J; Supplementary Fig. S5H). Taken collectively, our results pinpoint specific B-cell sublineages exhibiting increased proportions in diffuse-type gastric cancer and highlight epithelial cell–resident *KLF2* expression as a potential driver of plasma cell recruitment.

We further studied the relationship between epithelial cells from diffuse-type gastric cancers with pathways related to immune-cell biology. scRNA-seq–driven pathway analysis between diffuse- and intestinal-subtype epithelial cells revealed several upregulated genes in diffuse-type epithelial cells related to immune-signaling pathways, including numerous immunoglobulin (Ig) genes belonging to both light-chain and heavy-chain genes (IgG/IgA) and the Ig linker gene *JCHAIN* (Fig. 3K, top panel; Supplementary Table S11). A subcluster pathway analysis of upregulated genes revealed that most of these immune-related modules were expressed in EpiC cells, similar to *KLF2* (Fig. 3K, bottom panel; Supplementary Table S12). In contrast, downregulated genes did not show pathway enrichments related to immune signaling. Taken together, these data suggest a general transcriptional cassette expressed in diffuse-type gastric cancer epithelial cells related to engaging the tumor immune microenvironment.

### scRNA-seq Reveals Distinct Fibroblast Populations and *INHBA*–*FAP* Axis as a CAF Regulator

**Figure 4.**
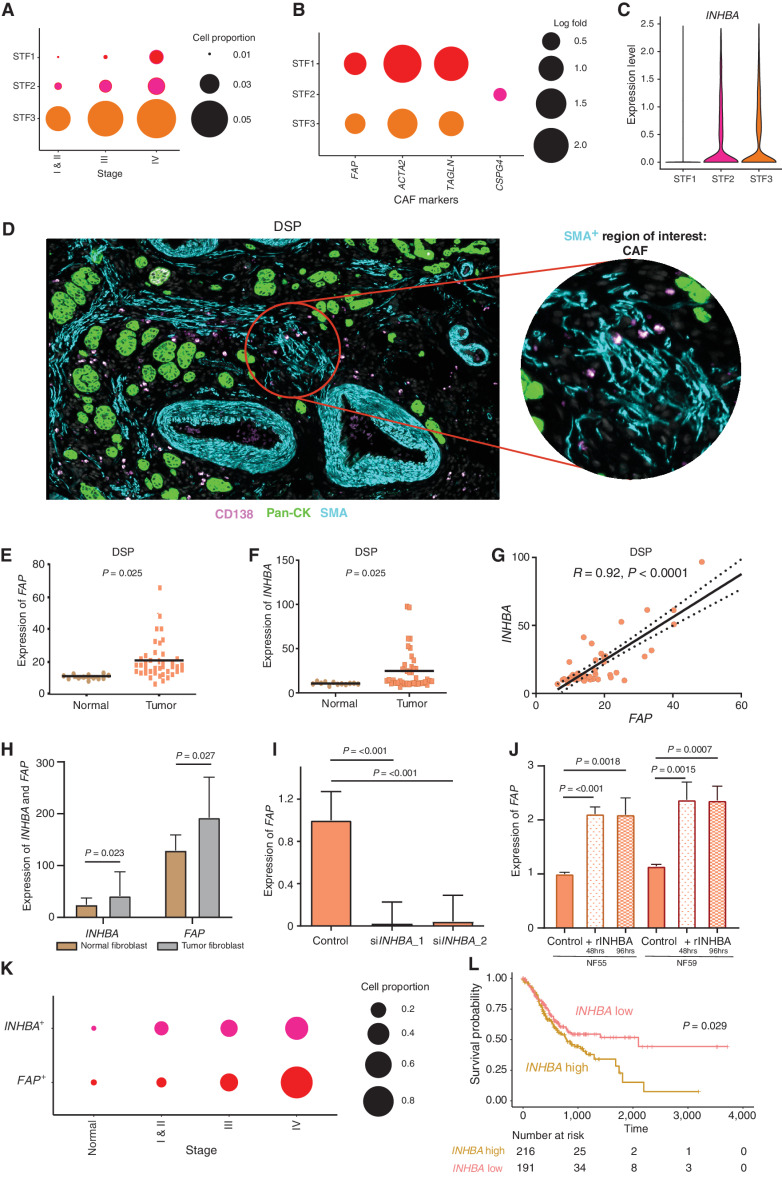
scRNA-seq enables identification of distinct gastric cancer fibroblast subtypes and *INHBA*–*FAP* axis as a CAF regulator. **A,** Bubble plots demonstrating stage-dependent increases in the proportion of fibroblast cells with STF3 being the dominant subcluster. The size of the circle represents the proportion of cells expressing subcluster-specific genes. **B,** Bubble plots showing fibroblast subclusters (STF1–3) expressing distinct CAF canonical markers (*FAP, CSPG4, ACTA2*, and *TAGLN*). The size of the circle represents the proportion of cells expressing different genes. **C,** Violin plot showing the expression of *INHBA* in STF2 and STF3 fibroblast clusters with negligible expression in the STF1 fibroblast cluster. **D,** Fibroblast ROIs captured by DSP analysis based on immunofluorescence staining for Pan-CK (epithelial, green), CD138 (plasma, pink), SMA (fibroblast, cyan), and DAPI (blue). The circular ROI is 300 μm in diameter. **E,** Bee swarm plot showing differential expression of *FAP* in fibroblast ROIs of normal and tumor samples by DSP (*n* = 13). **F,** Bee swarm plot showing differential expression of *INHBA* in fibroblast ROIs of normal and tumor samples by DSP (*n* = 13). **G,** Pearson correlation graph demonstrating strong positive correlations between *INHBA* and *FAP* gene expression in fibroblast ROIs using DSP. **H,** Bar graph showing significant expression of *FAP* and *INHBA* genes in flow-sorted tumor fibroblasts compared with matched normal fibroblasts (*n* = 10 each). **I,** Bar graph showing significant reduction in *FAP* gene expression after siRNA-mediated *INHBA* knockdown in tumor fibroblast lines. Two independent siRNAs were used. **J,** Bar graph showing significant increases in *FAP* gene expression in two normal fibroblast lines after treatment with recombinant INHBA (rINHBA) for 48 and 96 hours, respectively. **K,** Bubble plot depicting stage-dependent increases of *FAP*^+^ and *INHBA*^+^ cells in fibroblast cluster STF3 (*P* = 0.041). The circle sizes represent the relative proportion of cells expressing these genes. *P* values were computed using Kendall's τ method. **L,** Kaplan–Meier survival curves of TCGA-STAD data showing significant differences in overall survival between *INHBA*-high and *INHBA*-low samples. *P* values were computed using log-rank tests.

CAFs are known to influence tumor growth, migration, and invasion through the regulation of ECM components in various tumor types (44, 45). However, little is presently known about specific pathways of CAF regulation and heterogeneity in gastric cancer, due to limited *in vitro* models of gastric cancer CAFs and the challenges of deconvoluting bulk RNA-seq data (46). Using our scRNA-seq data, we first compared the tumor fibroblast clusters (STF1–3) according to clinical stage and histologic subtype. Supporting an important role for CAFs in gastric malignancy, we observed a stage-dependent increase in all three STF clusters, with STF3 as the dominant population (Fig. 4A). We next closely assessed a panel of CAF canonical markers, *FAP, CSPG4, ACTA2*, and *TAGLN* (Supplementary Table S4), in STF1–S3. Although STF1 and STF3 clusters exhibited upregulation of *FAP*, *ACTA2*, and *TAGLN*, the STF2 cluster exhibited upregulation of only *CSPG4* (Fig. 4B), further indicative of distinct CAF sublineages in gastric cancer.

TGFβ superfamily signaling has been reported to influence CAF function in other cancer types (47). We thus elected to study the activin–inhibin signaling module, a major component of the TGFβ pathway, comprising nine canonical genes (Supplementary Table S4). Among these nine genes, only *INHBA* exhibited significant upregulation in tumor-associated STF2 and STF3 clusters compared with normal (Fig. 4C). As tumor STF2 (*CSPG4*-high) and STF3 (*FAP*-high) represent distinct CAF populations, we then performed a coexpression correlation analysis between *INHBA* expression and the respective cluster markers. *INHBA* exhibited a significant positive correlation with *FAP* in STF3 (*R* = 0.21, *P* < 0.0001), whereas no correlations were found with *CSPG4* in STF2 (*R* = −0.03; Supplementary Fig. S6A). In TCGA bulk RNA-seq data, *INHBA* was similarly correlated to *FAP* (Pearson *R* = 0.59; *P* < 0.0001) but not to *CSPG4* (Pearson *R* = 0.00; *P* = 0.964; Supplementary Fig. S6B). To orthogonally support the association between *FAP* and *INHBA*, we adopted multiple approaches. First, we analyzed spatial DSP data of *FAP* and *INHBA* in fibroblast regions marked by α-smooth muscle actin (*n* = 13 samples: 4 ROI per sample; Fig. 4D). We observed higher expression of both *FAP* and *INHBA* in tumor fibroblasts compared with normal (*P* < 0.05; Fig. 4E and F), and within tumor fibroblasts there was a strong correlation between *FAP* and *INHBA* coexpression levels (*R* = 0.92, *P* < 0.0001; Fig. 4G). Second, in an independent cohort of 10 *in vitro* cultured patient-matched normal and tumor fibroblasts, isolated and purified by fluorescence-activated cell sorting from patients with gastric cancer (46), we confirmed *INHBA* upregulation (*P* = 0.023) in tumor fibroblasts, along with increased *FAP* (*P* = 0.027; Fig. 4H). Third, siRNA-mediated knockdown of *INHBA* in the tumor fibroblasts resulted in significant *FAP* gene downregulation (*P* < 0.001; Fig. 4I; Supplementary Fig. S6C). Conversely, treatment of two normal fibroblast lines with recombinant INHBA was sufficient to increase *FAP* expression at 48 (*P* < 0.001) and 96 (*P* < 0.001) hours (Fig. 4J). Taken collectively, these results highlight *INHBA* as a positive regulator of *FAP* in the gastric cancer STF3 fibroblast population.

We also studied correlations between *INHBA* expression and collagen-related gene expression (48–52), a surrogate for fibrogenic processes regulated by TGFβ signaling. Of nine collagen genes, five positively correlated with *INHBA*, including *COL1A1*, *COL1A2*, *COL6A3*, *COL8A1*, and *COL12A1* (*R* > 0.2, *P* < 0.0001; Supplementary Fig. S6D). To further verify these findings, we then analyzed DSP data and again confirmed a significant positive correlation of *INHBA* with *COL1A1*, *COL1A2*, and *COL6A3* (*R* > 0.69, *P* < 0.0001; Supplementary Fig. S6E; *COL8A1* and *COL12A1* were not represented on the DSP platform). These results support a positive relationship between *INHBA* activity and collagen gene induction. To explore the potential paracrine capability of *INHBA* (53), we then treated CAFs *in vitro* with recombinant INHBA for 48 hours and measured the expression of collagen genes. We observed significant increases in collagen genes, including TGFβ targets *COL1A1*, *COL1A2*, and *COL6A3* in two CAF lines (Supplementary Fig. S6F), consistent with a cell nonautonomous role for *INHBA*.

To assess the clinical relevance of our findings, we mapped *FAP* and *INHBA* expression levels across the gastric cancer cohort. We found an increasing abundance of *FAP*-positive and *INHBA*-positive cells in STF3, in a stage-wise manner from normal to stage IV (Fig. 4K; *P* = 0.041, after normalizing for differences in STF3 proportion). Survival analysis of TCGA samples by *INHBA* levels revealed a significantly poorer survival for tumor samples with high *INHBA* expression (HR: 0.70; 95% CI, 0.51–0.97, *P* = 0.029; Fig. 4L). This difference remained statistically significant even after adjusting for stage (HR: 0.71, *P* = 0.038). Similar findings were also seen in a pooled analysis of several available gastric cancer microarray data (ref. 54; *INHBA* high vs*. INHBA* low, HR: 0.80; 95% CI, 0.68–0.96, *P* = 0.014; Supplementary Fig. S6G).

### scRNA-seq of Gastric Cancer Organoids Supports Increased Cancer Cell Transcriptional Plasticity

**Figure 5.**
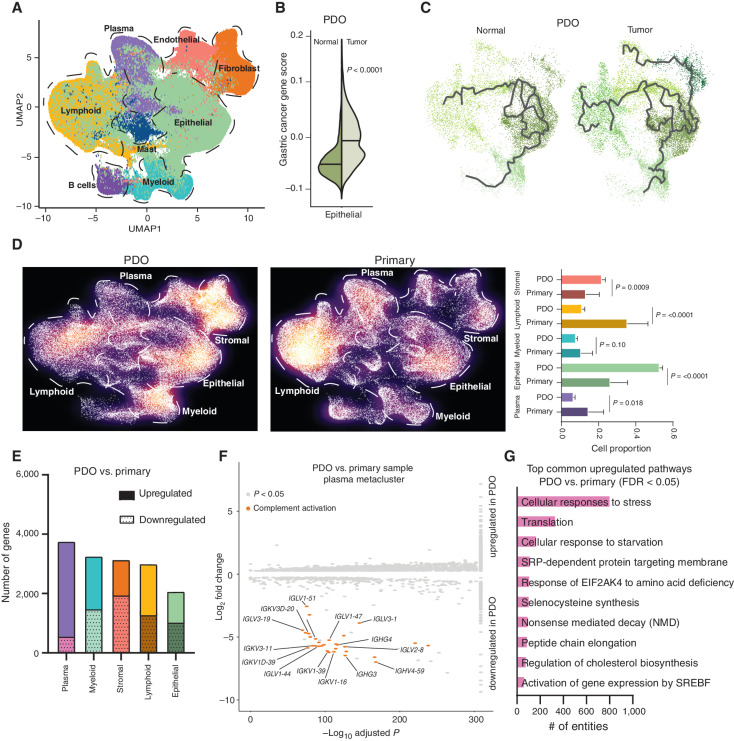
Comparative analysis of primary and organoid samples. **A,** UMAP representation of approximately 200,000 cells (∼48,000 cells from tumor PDOs with matched normal PDOs, combined with primary samples; ∼152,000 cells) recapitulating the major five metaclusters color-coded by their cell types. Each dot in the UMAP represents a single cell. **B,** Violin plot showing the expression of gastric cancer gene module scores in tumor PDOs compared with matched normal samples. **C,** Trajectory plot analysis of epithelial cells from tumor and normal PDOs demonstrating the expression of cellular differentiation gene programs in tumor PDOs depicted by long multiple branches. **D,** Density plot of UMAP representation comparing PDO and primary gastric samples demonstrating enrichment of lymphoid and plasma metaclusters in primary samples compared with PDOs. **E,** Graph showing the number of upregulated and downregulated genes in PDOs versus primary samples in the five metaclusters. The plasma meta-cluster shows the highest number of differentially expressed genes as compared with other metaclusters. **F,** Volcano plot of upregulated and downregulated genes in the plasma metacluster between PDOs and primary samples, showing significant downregulation of antibody-mediated complement factor genes in PDOs. *x*-axis shows the −log_10_ adjusted *P* value and *y*-axis log_2_ fold change in gene expression. **G,** Top common upregulated pathways in PDOs versus primary samples across all metaclusters.

Finally, PDOs are increasingly being used as a platform to model gastric cancer tumor behavior and drug responses (55–57). To investigate the extent to which *in vitro* organoid culture conditions affect transcriptional lineage states or overall cellular proportions compared with *in vivo* primary gastric cancers, we derived and examined four pairs of matched normal–tumor PDOs (*n* = 48,531 cells; <12 passages; Methods; Supplementary Fig. S7A). To perform a comparative cell-state analysis between PDOs and primary gastric cancers, we integrated the scRNA-seq data set from PDOs with the primary tumors. This analysis recapitulated the five major metaclusters (Fig. 5A). We then performed a sublineage-level analysis of epithelial and stromal metaclusters in the PDOs. For the PDO epithelial metacluster, the proportion of the three sublineages was EpiC (71% in PDO vs. 43% in primary), EpiInt (15% vs. 30%), and EpiPit (14% vs. 27%). In the PDO stromal clusters, we also observed pericytes (28% vs. 14% in primary), fibroblasts (32% vs. 45% in primary), and endothelial cells (39% vs. 37%; Supplementary Fig. S7B). These findings suggest that sublineage heterogeneity is indeed present in PDOs, although proportions may differ compared with primary gastric cancers.

Similar to primary gastric cancers, tumor PDO epithelial cells exhibited upregulation of cancer-associated modules and gastric cancer–related genes compared with normal PDO epithelial cells (*P* < 0.0001; Fig. 5B). Interestingly, trajectory plot analysis of epithelial cells in both normal and tumor PDOs demonstrated that epithelial cells from tumor organoids have multiple extended branches relative to normal epithelial cells, consistent with tumor PDO epithelial cells undergoing pervasive and ongoing differentiation/dedifferentiation (Fig. 5C). These findings are noteworthy given recent studies reporting that tumor-associated epithelial cells have increased transcriptional plasticity, which may drive key aspects of ITH (11). Our ability to observe similar phenomena in PDOs suggests that PDOs could be used as an *in vitro* experimental model to investigate molecular pathways governing transcriptional plasticity in gastric cancer.

Our analysis also revealed differences between primary samples and PDOs. For example, epithelial and stromal metaclusters in PDOs were significantly enriched, relative to lymphoid and plasma cell clusters that were depleted (Fig. 5D; *P* < 0.001 for stromal, epithelial, and lymphoid; *P* = 0.018 for plasma). A gene-expression comparison between PDO and primary samples indicated plasma cells as showing the greatest differences in gene-expression profile in PDOs, whereas epithelial signatures were relatively more conserved (Fig. 5E). In congruence with these findings, comparison of Reactome programs (58) between the PDOs and primary tumors by metaclusters confirmed a high overlap of Reactome programs for the epithelial [Jaccard similarity index (JSI) = 0.62] and stromal (JSI = 0.43) metaclusters, with poorer overlap for the plasma (JSI = 0.09) and lymphoid (JSI = 0.15) metaclusters. Pathway analysis of genes unique to plasma cells implicated “classical antibody-mediated complement activation” as significantly downregulated (Fig. 5F; Supplementary Table S13). Notably, a significant proportion of genes and pathways were also commonly upregulated in PDOs agnostic of cluster, which included “cellular response to starvation,” highlighting umbrella culture effects (Fig. 5G; Supplementary Table S14). Together, these results raise the possibility that besides epithelial cells, PDO culture may also influence the transcriptional profiles of other cell types associated with tumors.

## Discussion

**Figure 6.**
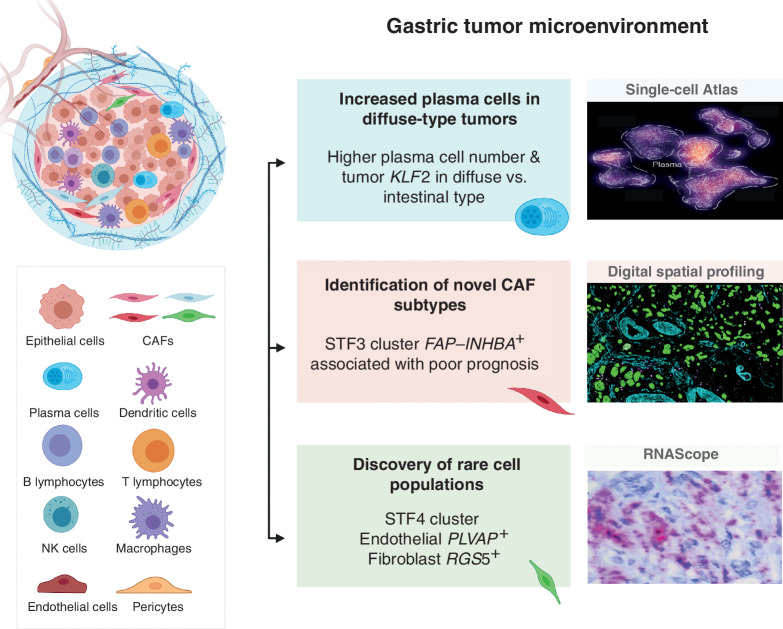
Comprehensive single-cell atlas of gastric cancer. This study included more than 200,000 cells from 31 primary gastric tumor samples. In total, 34 distinct cell-lineage states were identified, related by developmental trajectories and previously unreported rare cell populations. An increase in plasma cell proportions was observed as a feature of diffuse-type tumors associated with epithelial-resident *KLF2*. A stage-wise accrual of novel cancer-associated fibroblast subpopulations was marked by high *INHBA* and *FAP* coexpression. Findings were complemented using digital spatial transcriptomics and RNAScope. Our results provide a high-resolution molecular resource for gastric cancer translational studies, identifying intra- and interpatient lineage states across distinct gastric cancer subtypes.

In this study, we applied scRNA-seq across a large number of cells to discover several novel features of gastric cancer, including rare cell populations undergoing state transitions, cell type–specific expression programs associated with gastric cancer, and distinct plasma cell and CAF sublineages associated with gastric cancer histologic subtypes and clinical stages. Another notable aspect of our study was the application of very recently available spatial transcriptomics technologies (DSP) to orthogonally verify our key findings. Compared with earlier gastric cancer scRNA-seq studies, our experimental design and analyses are differentiated by a large cell number size (>200,000 cells, higher than all prior gastric cancer studies combined), samples reflecting multiple clinical stages and subtypes (*n* = 31 patients, 48 samples), and the comparative analysis of gastric organoids (Fig. 6). For example, Zhang and colleagues performed scRNA-seq on gastric cancer biopsy samples (*n* = ∼30,000 cells) from patients with premalignant lesions (atrophic gastritis and IM), and one early gastric cancer sample, focusing on the evolution of epithelial cells from normal to malignancy (14). At the terminal end of the cancer spectrum, Wang and colleagues performed scRNA-seq only on malignant ascites cells (*n* = ∼45,000 cells; ref. 11). Sathe and colleagues studied ∼55,000 cells from seven patients with gastric cancer and one patient with IM, generating a receptor–ligand network of the TME components, agnostic of tumor subtypes (13).

scRNA-seq analysis of tumor–normal comparisons performed in a cell-lineage–specific manner led to the emergence of a composite tumor profile assembled from distinct faulty signatures expressed by different lineages. Our results suggest that the greatest tumor-associated expression differences in the tumor epithelial component likely originate from chief cells and intestinal-type cells. Interestingly, high gastric cancer oncogene expression was observed in a specific subpopulation of intestinal-type epithelial cells (EpiInt1), suggesting that EpiInt1 cells likely represent a key epithelial cell population important in the transition into malignancy from metaplasia. One potential limitation of our study is the lack of directly inferred single cell–based DNA alterations, as currently available platforms are unable to deliver both single-cell DNA-level and RNA-level alterations from the same cell on a genome-wide scale. Additionally, the global clustering methodology used in our study does not preclude the possibility that more granular cell types may exist, which could be identified using refined local clustering (59, 60). Among nonepithelial cell types, lineage analysis of fibroblasts identified discrete CAF clusters, such as CAFs that were *LUM* positive or pericyte CAFs that were *CSPG* positive. Both *LUM* and *CSPG* encode proteoglycans, and pericytes are being increasingly recognized as key players in tumor vessel formation and growth (61). The differences in gene-expression profiles between these two fibroblast clusters may highlight distinct tumor-promoting functions. For example, *LUM*-associated CAFs may be involved in the proliferation and survival of tumor cells, whereas pericyte CAFs may be involved in neovascularization, thereby underscoring the functional heterogeneity of CAFs in gastric cancer. We also identified a small cluster of stromal cells (STF4) undergoing EndoMT—these cells expressed markers associated with both endothelial (*PLVAP*) and fibroblast (*RGS5*) lineages, confirmed after doublet filtering and by orthogonal RNAScope analysis. EndoMT, an embryonic process required for normal cardiovascular development (62), has been associated with TME plasticity, resistance to antineoplastic therapy, and TGFβ and BMP signaling (63–65). Together, these scRNA-seq findings suggest a conceptual model wherein individual cell lineages respond distinctly, either directly or indirectly, to malignant transformation, leading to a highly complex tumor ecosystem. It is reasonable to posit that these observations would have been largely obscured in bulk RNA-seq data.

CAFs, representing a predominant stromal cell population, have been shown to play cardinal roles in shaping tumor growth and metastasis in various tumor types (46, 66, 67). CAFs are thought to largely affect tumor behavior via ECM modeling, secretion of soluble factors, and promoting angiogenesis (66). In the case of gastric cancer, although some studies have investigated CAF-associated signaling pathways in tumor cells, molecular events underpinning CAF function and heterogeneity remain poorly defined. In our data set, we found that gastric cancer CAFs have at least three distinct subtypes (STF1–3), each expressing distinct subsets of canonical CAF markers. Moreover, *FAP*-high STF3 cells also exhibited high *INHBA* coexpression, a correlation we confirmed in multiple orthogonal settings inclusive of a clinical stage-wise gradation. Functionally, silencing of *INHBA* affected *FAP* levels in gastric cancer CAFs, implying a potentially direct role for *INHBA* in regulating *FAP* expression, and consistent with TGFβ signaling as a mediator of *INHBA* (68). A functional role for *INHBA* in gastric cancer CAFs has also been demonstrated by a recent study in gastric cancer that used bulk RNA-seq of laser-capture microdissection (LCM)–derived CAF samples (69). Clinically, patients with high *INHBA*-expressing tumors exhibited poorer survival outcomes in multiple gastric cancer cohorts, consistent with a tumorigenic function for INHBA (70–72). *INHBA* encodes a subunit of activin and inhibin, which have been reported to play opposing roles in many facets of normal biology and disease (73). It is possible that high *INHBA* expression levels may facilitate the formation of INHBA homodimers, otherwise known as Activin A, leading to the activation of activin receptors such as *ACVR1*, which has established roles in cancer (74). In other cancer types, aberrant increases in *INHBA* expression have been reported in both the epithelial and CAF components, involving autocrine and paracrine functions (75–78). Our observations put forth the INHBA pathway as a potential target to disrupt CAF function and warrant testing of drug modalities in appropriate model systems (79).

In most parts of the world, Lauren's histopathologic subtypes are frequently used as reference standards in gastric clinical pathology. Evolution of intestinal-type gastric cancers is usually ascribed to the Correa cascade, and the etiology and molecular features of diffuse-type gastric cancer are not well understood (80). In our study, scRNA-seq enabled lin-eage-based comparisons of the TME between these histologic subtypes, leading to the discovery of increased plasma cells in diffuse-type gastric cancers. Compared with T cells, there are fewer studies on B-cell and plasma cell populations in gastric cancer (81, 82). Derks and colleagues reported a higher proportion of tertiary lymphoid structures in genomically stable (GS) tumors with enrichment of B cells and CD4 T cells (83). One report by Katoh and colleagues indicated a finding of increased B-cell lineages (which includes plasma cells) in diffuse-type and GS gastric cancers using the TCGA data set and identified sulfated glycosaminoglycan as a key functional B-cell antigen in these tumors (84). Notably, B-cell/plasma cell infiltration has been associated with protumor (i.e., immune suppressive) and antitumor (i.e., immune active) growth in other cancers (85). For gastric cancer, we speculate a “protumor” function for plasma cell infiltration, because increased plasma cells are associated with diffuse-type gastric cancers that respond poorly to immune checkpoint inhibitors (86, 87) and exhibit classic “immune-suppressive” features (88). To identify mechanisms responsible for the plasma cell increase, we investigated KLF2, a transcription factor previously shown to modulate multiple myeloma cell adhesion and homing of plasma cells (40). We observed that *KLF2* expression in diffuse-type epithelial cells (EpiC cluster) was positively correlated with plasma cell proportions, and increased *KLF2* expression in diffuse-type and GS gastric cancers (Supplementary Table S15) in the TCGA cohort was consistent with a regulatory role for epithelial KLF2 in shaping plasma cell populations. We functionally explored this possibility in “humanized mice,” by xenografting human-derived diffuse-type gastric cancer cell lines that were *KLF2* positive and negative. We found that only tumors with *KLF2* had human plasma cell markers, implying a more casual role for epithelial *KLF2* expression in plasma cell recruitment. Using DSP, we also gained spatial insights of *KLF2* expression on primary samples and observed a trend for higher *KLF2* expression in epithelial cells proximate to plasma cells, further suggestive of a functional interaction between these cell types. The precise mechanisms underlying the plasma cell enrichment remain undefined. Earlier studies have reported that *KLF2* can modulate proinflammatory gene expression in monocytes and endothelial cells (89–91), and our findings suggest a role for KLF2 in driving tumor epithelial cell programs possibly involving paracrine cell signaling pathways (e.g., exosome-derived miRNAs; refs. 92–95). Interestingly, besides *KLF2*, we also found that epithelial cells in diffuse-type tumors (EpiC cluster) displayed a striking upregulation of multiple immune-signaling pathways, including Ig genes and *JCHAIN*, a gene responsible for the secretory form of Ig, mimicking gene-expression signatures of immune cells. This observation of an “epithelial-immune cell state” has been described in other cancers (96, 97). We report the existence of this phenomenon for the first time in diffuse-type tumors, adding a novel and singular dimension to gastric epithelial cell behavior. Probing the mechanisms underlying epithelial–plasma cell cross-talk in diffuse-type gastric cancers may pave the way for new intervention strategies to modulate TME for this recalcitrant subtype.

Among the platforms to study gastric cancer, PDOs have recently emerged as a promising system for *ex vivo* testing of therapeutic agents, precision oncology, and assessing driver gene function (98, 99). Our comparison of PDO-primary samples using scRNA-seq revealed that PDOs indeed maintained most major cell types, except for a depletion in lymphoid and plasma cell lineages. However, it should be noted that these PDOs represent a single snapshot, and assessment of organoid cellular compositions over time represents an area of future research. Global gene-expression analyses indicated the greatest changes in plasma cells compared with epithelial state. This suggests that PDOs may serve well to study gastric epithelial biology, whereas immune cells may be more affected by the process of culturing. These factors must be considered when using PDOs for personalized *ex vivo* drug testing, especially those involving immunologic pathways or characterizing TME differences. Interestingly, our data reconfirm the association of *ARID1A* loss with the induction of mucinous phenotype, an observation first made by Lo and colleagues (ref. 99; Supplementary Fig. S7C).

In conclusion, in one of the largest single-cell analyses of gastric cancer performed to date, our study forms a unique resource for generating novel biological insights on tumor cell types, subtype-based TME compositions, and cell–cell interactions in gastric tumors. In terms of future directions, our data support the need for further in-depth studies on plasma cell homing biology guided by epithelial KLF2 and the potential clinical implications of perturbing these interactions. The role of INHBA and the TGFβ superfamily in the regulation of CAFs also carries potential for therapeutic target and/or predictive biomarker discovery. We anticipate future work to utilize combinatorial single-cell approaches, including epigenetic, genetic, and transcriptional layers and spatial context to enhance our understanding of the gastric tumor architecture.

## Methods

### Ethics Declaration

The study was approved by the local ethics board (National Healthcare Group, Domain Specific Review Board Ref Nos: 2005/00440 and 2016/00059). All animal experiments and procedures were approved by the Institutional Animal Care and Use Committee (IACUC; IACUC #191440) of A*STAR in accordance with guidelines of Agri-Food and Veterinary Authority and the National Advisory Committee for Laboratory Animal Research of Singapore. Primary CAFs and normal fibroblasts (NF) were isolated from patients with gastric cancer who underwent gastrectomy without preoperative treatment at Kumamoto University after written informed consent was obtained from each patient. The study was approved by the Medical Ethics Committee of Kumamoto University (Approval Number: 1277). PBMCs were isolated from human blood obtained from healthy volunteers with written informed consent. Study protocols were approved by the SingHealth Centralised Institutional Review Board (reference number: 2017/2806). Protocols were performed in accordance with the Declaration of Helsinki for Human Research.

### Sample Acquisition and Tissue Processing

Patients diagnosed with gastric adenocarcinoma and undergoing surgical resection or endoscopy at the National University Hospital, Singapore, were enrolled after written informed consent was obtained. On-table endoscopic biopsies or surgical resection samples were harvested. For surgical samples, matched normal gastric tissues from sites displaced at least several centimeters from the tumor were used. Tissues from distant metastases to the peritoneum were taken during diagnostic laparoscopy. Tissues were collected in MACS tissue storage buffer (Miltentyi Biotec) immediately after biopsy or resection and stored on ice. Samples were processed using enzymatic and mechanical dissociation using a human tumor dissociation kit and the Gentle MACS Octodissociator (Miltentyi Biotec) following the manufacturer's “37_h_TDK_2” program. Dissociated cells were passed through a MACS smartstrainer (70 μm) and incubated with RBC lysis buffer for 5 minutes followed by PBS neutralization. All centrifugation steps were carried out at 300 × *g* for 7 minutes. Dissociated cells were washed twice in PBS + 1% bovine serum albumin (BSA) and filtered through a 40-μm smartstrainer. Live-cell counts were obtained by manual cell counting using 1:1 trypan blue dilution. Cells were concentrated to 800–1,200 live cells/μL and then processed for single-cell analysis.

### Single-Cell Sequencing

Samples from each patient were processed in a single batch for library preparation. The Chromium Single-Cell 5′ Library and Gel Bead Kit (10× Genomics) was used according to the manufacturer's protocols. Briefly, gel bead-based emulsions (GEM) were generated by combining barcoded single-cell 5′ Gel Beads, cells, and partitioning oil. Ten times barcoded, full-length cDNAs generated from GEMs were amplified by PCR. Enriched libraries were enzymatically digested, size selected, and adaptor ligated for sequencing. To obtain a TCR repertoire profile, VDJ enrichment was carried out as per the Chromium Single-Cell V(D)J Enrichment Kit, Human T-cell #1000005 (10× Genomics) using the same input samples. Sequencing libraries were generated with unique sample indices for each sample and quantified using the Kapa library kit. Quantified libraries were sequenced on an Illumina Hiseq4000 sequencer.

### Bioinformatic QC, Normalization, Clustering, and Differential Gene Expression of Single-Cell Data

Cellranger v3.0 (https://support.10xgenomics.com/single-cell-gene-expression/software/) was used to align FASTQ sequencing reads to the hg38 reference transcriptome, generating single-cell feature counts for each sample. Using Seruat version 3.0 (100), each sample was considered for genes/features shared by three or more cells, and cells showing 500 or more features and fewer than 6,000 features. Cells with mitochondrial RNA percentages of >20 were filtered out. SCtransform normalization was performed on each sample data set separately, along with regression of mitochondrial RNA as a variable. Single-cell data sets were combined by first developing an initial reference data set of 18 gastric samples with high cell numbers encompassing different characteristics of the primary and peritoneal metastasis samples for identifying anchors between pairs of samples and subsequently integrating the remaining samples as “query” data sets (18). Subsequently, PrepSCTIntegration was run to select features for downstream integration and FindIntegrationAnchors to identify anchor genes. The integrated data were scaled, and principal component analysis was performed. Data were visualized using Uniform Manifold Approximation and Projection (UMAP). Cell clusters were identified by a shared nearest-neighbor (SNN) modularity optimization-based clustering algorithm set at a resolution of 1. To identify differentially expressed genes for cluster demarcation, the FindAllMarkers module was used, and genes expressed in more than 25% of the cells in each cluster were selected. Differentially expressed genes from each cluster were compared with sets of previously described cell-type markers to assign cell identities, lineages, and sublineages. The Addmodulescore function was used to calculate composite module scores for different gene-expression programs in each single cell. These scores were calculated by averaging the expression levels of all the genes in each program at the single-cell level, subtracted by the aggregated expression of randomly selected control feature sets. Supplementary Table S2 lists the genes used for the various gene-expression programs/modules. For organoid analysis, the reference 18 scRNA sample data set (above) was integrated with the remaining primary, peritoneal, and organoid data sets, resolving cell clusters by SNN clustering at a resolution of 1.25. Organoid data were integrated with primary sample data sets using canonical correlation analysis (100). Pathway analysis was performed using the REACTOME database (https://reactome.org/) separately for differentially upregulated and downregulated genes (adjusted *P* < 0.05) for each cluster. Pathways with entities *P* value and FDR < 0.05 were investigated. Using these pathways, an overlap analysis was performed between epithelial metacluster and sublin-eages. For individual reclustering of metaclusters, we used LIGER (101), which relies on integrative nonnegative matrix (iNMF) factorization to identify shared and data set–specific factors. Briefly, we first selected variable genes, scaled them without centering followed by iNMF and quantile normalization of factors using the selectGenes, scaleNotCenter, optimizeALS (*k* = 30), and quantileAlignSNF modules of LIGER. These iNMFs were added to Seurat for UMAP and clustering visualization.

### Doublet Analysis

scRNA-seq data are commonly affected by technical artifacts known as “doublets,” which limit cell throughput and lead to spurious biological conclusions such as discovery of mixed linages. DoubletFinder was used to identify doublets using gene-expression data (29). DoubletFinder predicts doublets according to each real cell's proximity in gene-expression space to artificial doublets created by averaging the transcriptional profile of randomly chosen cell pairs. After identifying doublet cells, these were removed from the data set.

### Molecular Subtyping

TCGA molecular subtyping was performed as previously described (102). Briefly, gastric cancers were first classified as Epstein–Barr virus (EBV) or microsatellite instability (MSI) using EBV-encoded RNA *in situ* hybridization (EBER–ISH) and mismatch-repair (MMR) protein IHC.

#### MMR IHC

Tissues were cut into 4-μm-thick sections. The MMR panel consisted of monoclonal antibodies—mouse anti-MLH1 (Ventana, M1), mouse anti-PMS2 (Ventana, A16-4), mouse anti-MSH2 (Ventana G219-1129), and rabbit anti-MSH6 (Ventana, SP93). The panel was performed using the Ventana Optiview DAB Detection kit, the Ventana Optiview Amplification Kit, and ancillaries on the fully automated Roche Ventana Ultra instrument. MMR loss was determined when the tumor showed loss of expression for the examined MMR proteins. Normal tissue adjacent to the tumor was used as a positive internal control.

#### EBER–ISH

Tissues were cut on 4-μm-thick sections. The EBER probe is a fluorescein-conjugated oligonucleotide ready-to-use ISH probe from Leica Bond, and with hybridization for 2 hours. Detection was performed with anti-FITC antibody, Leica Bond DAB polymer kit, and ancillaries on the fully automated Leica Bond III Biosystem. EBER positivity was determined by localization of the EBER signal within the nucleus of the tumor cells.

WES of the remaining samples was performed as previously described (103). Briefly, 100 ng of DNA from each sample was sheared, adapter ligated, exome captured using the Agilent Sureselect Human all exon kit v6, and sequenced using Illumina Hiseq4000 platform. Tumors were then classified as either chromosomal instability (CIN) or GS based on the copy-number profiles inferred from WES using GISTIC2 (104) or scRNA-seq. Tumors were clustered with a uniformly processed in-house data set of >200 gastric cancers, using Euclidean distance and Ward method, based on thresholded copy numbers from significantly altered sites identified by GISTIC2 (*q* < 0.05). Gastric cancer samples exhibiting elevated copy-number changes were assigned as CIN. We confirmed significant associations between GS tumors with diffuse histologic subtype (*P* = 0.03; Chi-square test), and increased T-cell infiltrates in MSI-positive tumors (*P* = 0.021). Samples with whole-genome doubling predicted by WES (thus classified as CIN) also exhibited high CNVs measured by scRNA-seq.

### Trajectory Analysis

Trajectory analysis was performed with Monocle 3.0 using two approaches (105, 106). For plasma cell pseudotemporal analysis, plasma cells were extracted from the data set. Monocle was used to preprocess, align, perform UMAP directionality reduction and cell clustering, and to develop learned graphs and cell orders. To compare trajectories between normal and tumor cells, the Seurat wrapper for Monocle 3.0 was used to cluster cells and develop learned graphs. To assess the robustness of inferred trajectories, we performed bootstrapping by randomly downsampling samples by 10% and computing binomial *P* values for each major branching node.

### CNV Analysis

To identify large-scale chromosomal CNVs, InferCNV (https://github.com/broadinstitute/inferCNV) was used to explore tumor scRNA-seq compared with normal scRNA-seq data sets, and between diffuse-type and intestinal-type scRNA-seq data sets. Expression intensities in epithelial cells of genes across positions of the genome were compared with a set of reference T and B cells. Genes with a mean count number of 0.1 or less across cells were excluded. In addition, the hidden Markov method (HMM; “type i6”) was used, applying noise filters and “subcluster” analysis. For each sample, the gene expression of cells was restandardized and values were limited from −1 to 1. As the HMM model uses values that are not centered on one but six states, to make gains and losses symmetrical, we merged all “state 6” states with “state 5” before restandardization. The final CNV score of each cell was calculated as the sum of the absolute values of each CNV region. We also used CONICSmat for CNV calling (107). Briefly, after filtering out uninformative genes a normalization factor was calculated for each cell and the average expression in each cell was centered using the calculated normalization factor. The z-score of the centered gene expression across all cells was calculated. Based on these z-scores, a Gaussian mixture model was calculated to determine regions showing an average gene-expression bimodal distribution across cells. Only results for regions harboring more than 100 expressed genes were calculated to ensure the predictions were not influenced by a few differentially expressed genes in a small region. To avoid batch effects, we called CNV for each sample separately on the gene-expression matrix on all cells.

### TCR Analysis

Cellranger v6.0 was used (using mkfastq module) to align FASTQ sequencing reads to the hg38 reference transcriptome. To generate single-cell V(D)J sequences and annotation for each sample, we used cellranger vdj module. Basic statistics and diversity index were estimated using immunarch (v0.6.6; https://immunarch.com/index.html).

### DSP and Analysis

Gastric cancer and normal FFPE tissues were mounted on super frost slides to validate the spatial profiling of RNA by the NanoString GeoMx DSP platform. For H&E staining, FFPE slides were deparaffinized with histoclear, rehydrated and stained with Hematoxylin Solution. Slides were counterstained with eosin and mounted with mounting media. FFPE slides were subjected to conventional tissue preprocessing (deparaffinization and rehydration). For RNA profiling, the transcriptomics cancer atlas with a 1,800-plex RNA probe set was used along with an additional set of 12 genes (Supplementary Table S16). Standard fluorescence-labeled morphology marker panel consisting of Pan-CK for epithelial regions, CD138 for plasma cells, α-smooth muscle actin for fibroblast and nuclear stain were used as ROI selection references. Twelve ROIs (four for each region) measuring 300 μm in diameter on each slide were drawn and selected. All oligos from the selected ROI were collected into 96-well plates using the proprietary UV-guided technology in the DSP approach. Resultant oligos representing individual targets for individual ROIs were sequenced using Illumina sequencing. Data were analyzed by uploading the counts data set from the Illumina run into the GeoMx DSP analysis suite. Biological probe QC was performed using default settings. Scaling was performed using geometric means and normalization using Q3 averages of housekeeping genes.

### Humanized Mouse Model

Details of the humanized mouse model experiment have been described previously (108). One- to three-day-old NSG pups were sublethally irradiated at 1 Gy and engrafted with 1 × 10^5^ human CD34^+^ cord blood cells (HLA-A24:02; STEMCELL Technologies) via intrahepatic injection. Mice with more than 10% human immune-cell reconstitution (calculated based on the proportion of human CD45 relative to the sum of human and mouse CD45) were included in the study. In total, two diffuse-type gastric cancer cell lines of HLA-A24:02 subtype were selected for the experiment (*KLF2* positive, GSU; *KLF2* negative, SNU1750; refs. 109, 110). For each cell line, five humanized mice and five NSG mice were injected with the tumor cells and observed for one month. Mice were sacrificed at the end of one month, necropsies were performed, and tumors were harvested and sequenced (bulk RNA-seq).

### IHC for IRF4

IHC analysis was performed to evaluate the expression of IRF4 in diffuse and intestinal gastric cancer samples using MUM1 (IRF4) primary antibodies (Clone MUM1p, DAKO, IS64430). IHC stains of 4-μm paraffin sections of patient samples were performed using a Bond Max automated immunostainer (Leica Biosystem) using antibodies for IRF4 (MUM1p, 1/500; Dako). Briefly, sections were deparaffinized and rehydrated followed by antigen retrieval at pH 9.0 (Tris buffer) for 36 minutes. Sections were treated with peroxidase solution followed by incubation with MUM1 primary antibody for 60 minutes according to the manufacturer's protocol. Tissue sections were further incubated with horseradish peroxidase–labeled polymer secondary antibody, and localization was performed with horseradish peroxidase–labeled polymer with DAB using a bond polymer refine detection kit (Leica Bondmax, Leica Biosystems) according to standard protocols. Appropriate positive controls were immunostained in each batch of IHC. Gastric epithelial cells served as an internal negative control. MUM1 expression was predominantly observed in the nuclei of plasma cells along with some weak expression in cytoplasmic regions. Strong nuclear expression was considered as positive staining, whereas standalone cytoplasmic staining was considered negative. For each case, 10 fields (at 20× magnification) were selected randomly, and MUM1-positive cells were counted in each field using the ImageJ software. A mean value of positive MUM1 expression was calculated for each case.

### Fibroblast Cell Culture Experiments

Human gastric fibroblast cell lines were derived from surgical gastric tissue of patients with gastric cancer who underwent gastrectomy without preoperative treatment with written informed consent from each patient. CAFs were established from the tumoral gastric wall, and NFs were from nontumoral gastric wall according to previously established protocols (111, 112). Fibroblast cell lines were cultured in RPMI 1640 containing 10% FBS (normal medium) and incubated at 37°C with 5% CO_2_. *INHBA* expression was downregulated by transfecting cells with predesigned Silencer Select siRNAs directed against *INHBA* (#1, s7434; #2, s7436; catalog no.4427037; Thermo Fisher Scientific) and a nontargeting siRNA (#4390843; Thermo Fisher Scientific) was used as the negative control. CAFs were transfected with annealed siRNAs for 48 hours (5 μmol/L) using Lipofectamine RNAiMAX (#13778-150, Thermo Fisher Scientific). Real-time PCR primers used were:

*INHBA*: F 5′ AGCTCAGACAGCTCTTACCACA 3′; R 5′ TTTTCCTTCTCCTCTTCAGCA 3′
*FAP:* F 5′ TGGCGATGAACAATATCCTAGA 3′; R 5′ ATCCGAACAACGGGATTCTT 3′

NFs were seeded and incubated at 37°C with 5% CO_2_. After 24-hour seeding, the cultured cells were treated with 100 ng/mL recombinant human Activin A (BioLegend) for 48 and 96 hours, respectively. Collagen genes were measured in recombinant INHBA-treated and control fibroblast lines using the following primers:

*COL1A1*: F 5′ AGACGAAGACATCCCACCA 3′; R 5′ GTCATCGCACAACACCTTGC 3′
*COL1A2*: F 5′ CTGGAGAGGCTGGTACTGCT 3′; R 5′ GAGCACCAAGAAGACCCTGA 3′
*COL6A3*: F 5′ ACCGTCCAACAGGTCATCTC 3′; R 5′ CTCTTGCCACCAACACCTGG 3′

All experiments were performed in triplicate. Data are presented as mean ± standard deviation (SD). Statistical significance was determined using a two-tailed Student *t* test.

### *KLF2* Knockdown Model

LMSU cells were transfected with pooled siRNAs for *KLF2* [Dharmacon, L-006928-00-0020 ON-TARGETplus Human *KLF2* (10365) siRNA-SMARTpool] or negative control siRNAs (Dharmacon, D-001810-10-20 ON-TARGETplus Nontargeting Control Pool) at 100 nmol/L using Lipofectamine 2000 reagent (Thermo Fischer Scientific, 11668027). To obtain stable knockdown of *KLF2* in GSU cells, cells were plated at a concentration of 500,000 cells in a 10-cm dish and infected with MISSON lentiviral transduction particle encoding PLKO.1-PURO-CMV-tGFP-KLF2 (clone ID TRCN000418423) or GFP (as nontargeting control). After 48 hours, transfected cells were selected with 2 μg/mL puromycin. The transfected cells were then expanded and selected in culture medium plus puromycin (1 μg/mL) for 3 weeks to obtain stable *KLF2*-knockdown cells. Total RNA was extracted using the Qiagen RNAeasy mini kit according to the manufacturer's instructions. RNA was converted to cDNA using Improm-II Reverse Transcriptase (Promega) or iScript CDNA synthesis kit (Bio-Rad). Quantitative PCR was performed in triplicate using Quantifast SYBR Green PCR kit (Qiagen) on an Applied Biosystems HT7900 Real-Time PCR System for 40 cycles using the following primers with an annealing temperature of 55°C:

*KLF2*: F 5′CGGCAAGACCTACACCAAGAGT 3′ R 5′CGCACAGATGGCACTGGAATG 3′
*ACTB:* F 5′ CATGTACGTTGCTATCCAGGC 3′; R 5′ CTCCTTAATGTCACGCACGAT 3′

Fold change was calculated using the Delta–Delta Ct method. Total intracellular protein was extracted with RIPA lysis buffer (Thermo Fisher). Protein concentration was determined using a bicinchoninic acid (BCA) assay kit (Thermo Fisher).

### Western Blotting

Equal amounts of protein from each sample were separated on 4% to 12% sodium dodecyl sulfate polyacrylamide gel (SDS-PAGE) and electrotransferred onto polyvinylidene difluoride (PVDF) membranes. Membranes were blocked with 5% fat-free dry milk in 0.1 M Tris-buffered saline–0.1% Tween-20 buffer (TBST) for 1 hour. The membranes were sequentially incubated with a KLF2 primary antibody (Affinity Bio; dilution of 1 in 1,000) or GAPDH primary antibody (Proteintech; dilution of 1 in 3,000) overnight and horseradish peroxidase–conjugated secondary antibody (anti-rabbit and anti-mouse, Santa Cruz Biotechnology) for 1 hour. Blots were developed with an enhanced chemiluminescence reagent (Amersham Biosciences) and quantified by densitometric scanning and analyses using a ChemiDoc system (Bio-Rad).

### Transwell Migration Assay

Transwell migration assays were performed with human plasma-blasts/plasma cells isolated from primary human PBMCs or KMS-11 cells, a human multiple myeloma cell line. Briefly, human plasma-blasts/plasma cells from PBMCs were isolated using the Plasma Cell Isolation Kit II according to the manufacturer's instructions (cat # 130-093-628; Miltenyi Biotec). The purity of isolated plasmablasts/plasma cells was assessed by flow cytometry using CD19 and CD38 antibodies (BD Biosciences), whereby peripheral blood plasmablasts/plasma cells were defined as CD19^+^ CD38^+^ cells. We loaded 2 × 10^4^ cells per well into the top chamber, and for KMS-11 cells, 2.5 × 10^5^ cells were resuspended in 200 μL of the migration buffer and loaded onto the upper chamber of transwell inserts (Transwell Permeable Support with a 5.0-μm polycarbonate membrane, 6.5-mm insert; 3421, Corning). Gastric cancer cells were resuspended in 600 μL of migration buffer (0.5% BSA-RPMI 1640) and seeded into the bottom chamber of 24-well transwell plate (catalog no. 3421; Corning) and allowed to settle for 4 to 6 hours. Recombinant human CXCL12/SDF1a (350-NS-010-CF; R&D Systems) resuspended in 600 μL migration buffer (final concentration of 200 ng/mL) or migration buffer alone were used as positive and negative controls, respectively. Twenty to 24 hours later, unmigrated cells in the top chamber were counted. Unmigrated cells were subtracted from total cells seeded, and the proportion of migrated cells was calculated. Pair-wise comparisons were performed using the Mann–Whitney test.

### RNAScope

RNAScope ISH in gastric cancer samples was performed according to the manufacturer's protocols (113). Briefly, FFPE slides were baked at 60°C for 1 hour before being deparaffinized in xylene and 100% ethanol. After drying slides for 5 minutes at room temperature, H_2_O_2_ was added for 10 minutes at room temperature. For antigen accessibility, slides were incubated in boiling antigen retrieval solution (<98°C) for 15 minutes, washed in water twice, dehydrated in 100% ethanol, and finally treated with Protease Plus for 30 minutes at 40°C. Probes were then hybridized for 2 hours at 40°C followed by RNAScope amplification and chromogenic detection. RNA scope 2.5 HD Duplex detection kits were used for simultaneous visualization of two RNA targets in single-cell resolution using Hs-PLVAP HRP-GREEN (437461) and Hs-RGS5-C2 AP-FAST RED (533421; ACD bio). Sections were counterstained with hematoxylin and mounted with Vectamount. Duplex probes targeting CI-PPIB and C2 -POLR2A (322435) were used as positive control probes and dihydrodipi-colinate reductase (dapB), a bacterial gene (310043) as a negative control probe

### Analysis of Bulk RNA-seq Data

To assess cellular abundances in bulk tissue transcriptome profiles, CIBERSORTx was used to estimate cellular abundances of B cells and plasma cells in intestinal and diffuse TCGA-STAD data sets downloaded from FireBrowse (9, 114). For reference signature gene matrices, non–small cell lung cancer (NSCLC) PBMC scRNA-seq data sets provided with the CIBERSORTx suite were used. To compare single-cell RNA data with gastric bulk RNA data, combined TCGA and GTEx gene-expression data sets were used from the Xena browser (115). For survival analysis of *INHBA*, pooled survival analysis of several available gastric cancer microarray data sets was performed using the Kaplan–Meier plotter tool, gastric cancer subgroup (https://kmplot.com/analysis/index.php?*p*=service&cancer=gastric). Both *INHBA* probes (Affy ID: 204926_at and 210511_s_at) were pooled, and the mean expression of both probes was used to generate *INHBA* high and low subgroups by dividing samples at the median. For the survival analysis of *INHBA* in TCGA, samples were divided in *INHBA* high and low at the median value, and adjustment of stage was performed using Cox regression.

### Analysis of *KLF2* cis-Regulation

H3K27ac ChIP-seq and RNA-seq of 24 primary gastric cancer samples were performed and analyzed as previously described (116). To explore the *KLF2* locus in greater detail, we first identified *KLF2* promoter regions using an in-house gastric cancer promoter catalog derived from the H3K27ac ChIP-seq of 26 in-house gastric cancer cell lines. For each primary gastric cancer sample, the input subtracted H3K27ac signal at the *KLF2* promoter region was then computed using bigWigAverageOverBed to yield reads per kilobase per million.

### Generation, Maintenance, and Single-Cell Sequencing of Gastric Cancer PDOs

Human gastric tissues were biopsied from tumor and matched adjacent normal sites of each patient during surgical intervention. Tissues were processed as previously described (117). Briefly, tissues were minced and washed in Dulbecco's phosphate-buffered saline (DPBS; Thermo Fisher Scientific) and digested in DPBS containing 1 mg/mL collagenase (Sigma-Aldrich) and 2 mg/mL BSA (Sigma-Aldrich) for 30 minutes at 37°C. Digested tissues were passed through 30-μm filters (Miltenyi Biotec). Filtered cells were pelleted at 300 × *g* for 5 minutes, resuspended in Matrigel (Corning Life Sciences), and seeded into multiwell plates (Thermo Fisher Scientific). Cultures were maintained in custom gastric PDO culture medium at 37°C in 5% CO_2_ and monitored daily for organoid generation. The culture medium in each well was replaced with fresh medium on alternate days. PDOs were passaged once every 7 to 10 days at a 1:3 ratio. The median establishment time to the respective passage at time of sequencing was 17 weeks (range: 17–30 weeks; passage numbers: 9–11). Gastric organoids were harvested from gel matrices by washing briefly with DPBS, incubating with Trypsin-EDTA at 37°C for up to 30 minutes, and pelleted at 300 × *g* for 5 minutes. Supernatants were discarded, and cell pellets were washed twice with 10 mL DPBS each and filtered through cell strainers (mesh size: 30 μm). After centrifugation at 300 × *g* for 5 minutes, the supernatant was discarded, and cells were washed with 1× DPBS and then resuspended at ∼1,000 cells/μL in 1× PBS containing 0.4% BSA. Organoid scRNA-seq libraries were prepared using the 10× Genomics Single-Cell 3′ Gel Bead and Library Kit.

### Statistical Analysis

All analyses were done using R (V.4.0.3) with statistical significance set at *P* < 0.05 adjusted for multiple testing. Wilcoxon rank sum test was used to evaluate associations with continuous variables. Student *t* test was used to evaluate associations with parametric continuous variables. Bivariate correlation analysis was performed using Pearson or Kendall's Tau (clinical stage correlation). Significance of overlapping CNVs called by WES and scRNA-seq was assessed using hypergeometric distribution tests by phyper (lower.tail = FALSE) in R (V.4.0.3). Kaplan–Meier curves with log-rank statistics were used to compare overall survival. Pearson correlation analysis for TCGA bulk RNA-seq was performed using cBioPortal. We computed JSI for the top 30 significant Reactome programs (58) between PDOs and primary tumors by their metaclusters.

### Data Availability

scRNA-seq data have been uploaded to the Gene Expression Omnibus repository: https://www.ncbi.nlm.nih.gov/geo/query/acc.cgi?acc=GSE183904.

## Supplementary Material

Supplementary Data

Supplementary Data
