# Supplementary material for: Single-Cell Atlas of Lineage States, Tumor Microenvironment, and Subtype-Specific Expression Programs in Gastric Cancer
Source: Cancer Discov. 2022 Mar 8;12(3):670–91. doi: 10.1158/2159-8290.CD-21-0683 (PMC9394383; doi:10.1158/2159-8290.CD-21-0683)
Supplement: Supplementary Data [file cd-21-0683_supp.pdf]

Supplementary Figure 1

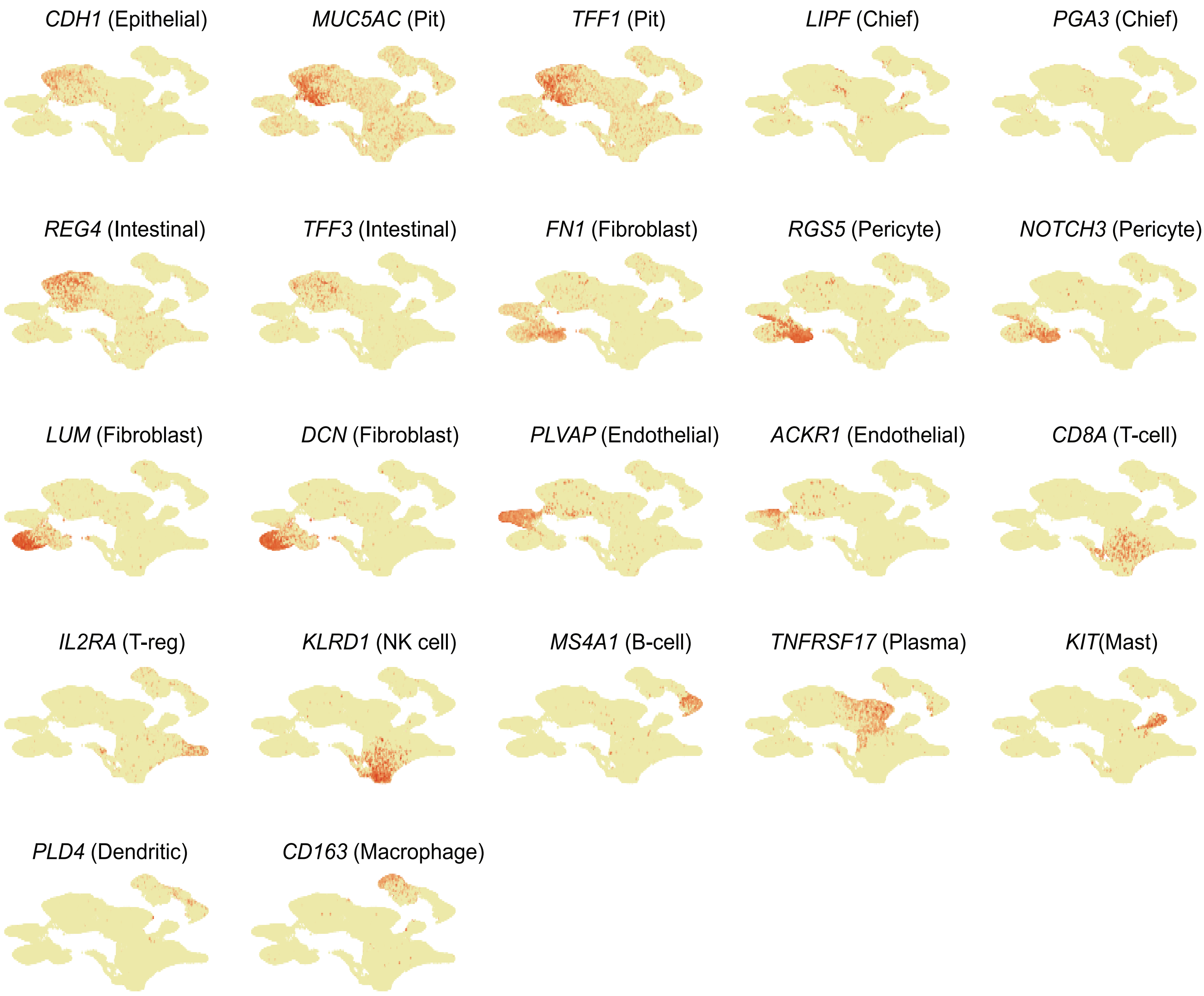

Supplementary Figure 1. UMAPs depicting cell lineages marked by genes specific to each cluster.

The epithelial cell meta-cluster is marked by *CDH1* expression and the different subclusters of epithelial cells such as pit mucous cell (Pit) marked by *MUC5AC*, *TFF1*, chief cells (Chief) marked by *LIPF* and *PGA3* expression and intestinal cells (Intestinal) marked by *REG4* and *TFF3* expression. Other cell lineages such as fibroblast are marked by the expression of *FN1*, *LUM*, *DCN*, pericytes marked by *RGS5* and *NOTCH3* expression, endothelial cells marked by *PLVAP*, *ACKR1*, T cells marked by *CD8A*, T regulatory cells (Treg) marked by *IL2RA*, NK cells marked by *KLRD1*, B-cell marked by *MS4A1*, plasma cell marked by *TNFRSF17*, mast cell marked by *KIT*, macrophages marked *CD163* and dendritic cells marked by *PLD4* expression.

Supplementary Figure 2

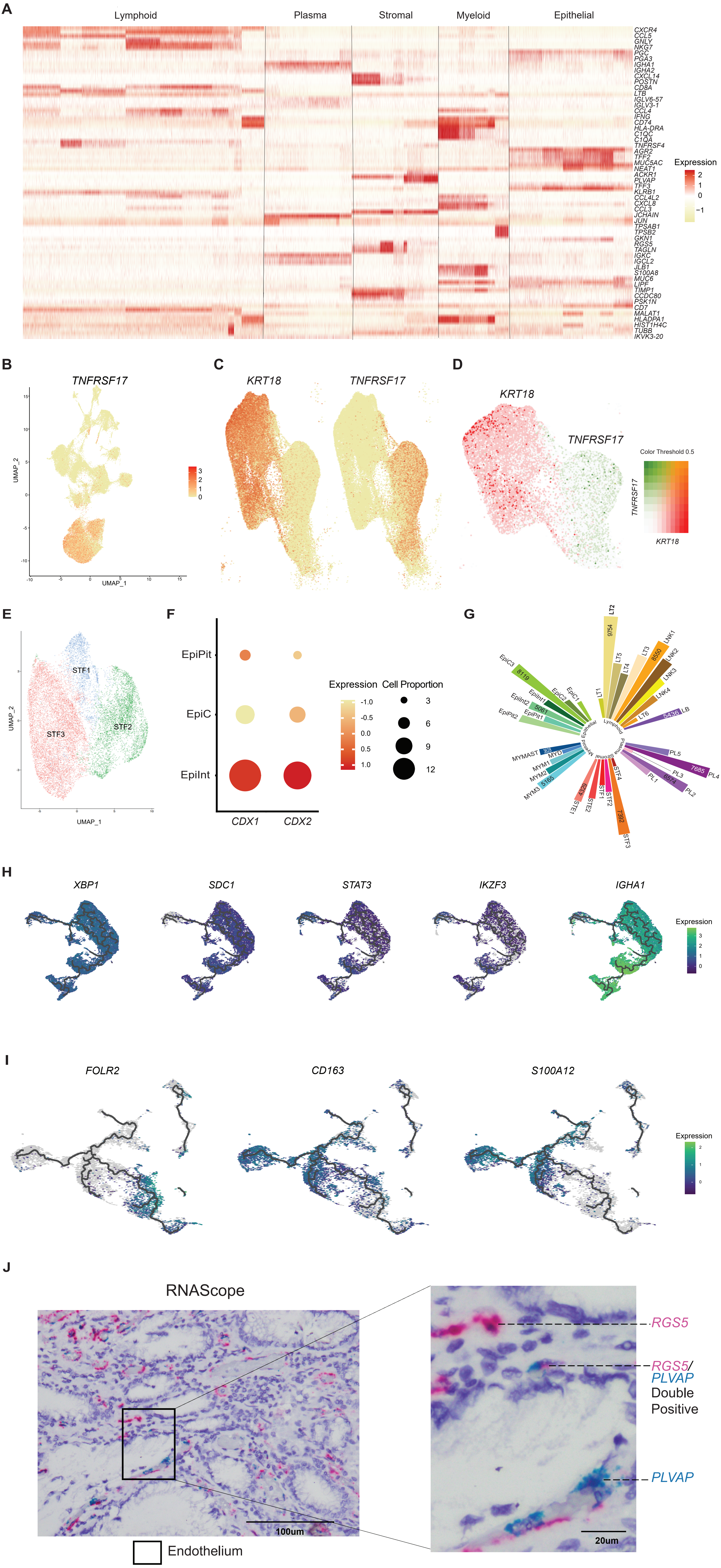

Supplementary Figure 2. Summary of cell-type markers, relative proportion of cells, and trajectory analysis.

A. Cell-type markers. Heatmap based on top 3 differentially upregulated genes for each cluster. The relative expression of genes across the cells is shown, sorted by cell type. The cell type markers were identified in an unbiased fashion (Wilcoxon rank-sum test, adjusted p value < 0.05). The top gene of each cluster is labelled.

B. UMAP depicting reclustering of epithelial and plasma cells using integrative Non-Negative Matrix Factorization (iNMF) reconfirms separation of plasma cells into an independent cluster, separate from epithelial cells.

C. Feature plot depicting epithelial and plasma meta-clusters with *KRT18* (epithelial cell) and *TNFRSF17* (plasma cell) markers in red. The two canonical markers show very minimal overlap between the epithelial and plasma clusters.

D. Blended feature plot of *KRT18* and *TNFRSF17* in epithelial and plasma cells. Co-plotting of *KRT18*-only marked in red, *TNFRSF17*-only marked in green, and *KRT18/TNFRSF17* dual-positive cells in orange. Epithelial cells and plasma cells are distinct due to the lack of dually-marked cells.

E. UMAP depicting reclustering of cancer-associated fibroblasts. There is strong correlation between global clustering (involving all cells), with weighted average overlap between global and subtype clustering of 0.81, p <0.0001.

F. Bubble plot showing expression of *CDX1* and *CDX2* genes in epithelial subclusters with EpiInt demonstrating major expression. The size of the bubble represents the percentage of cells expressing these genes, while the color represents the average expression of the gene.

G. Circular bar plot depicting the relative proportion of cells across different meta-clusters and their respective subclusters. The absolute cell numbers of the top two subcluster under each meta-cluster are marked within the bar.

H. Trajectory analysis of plasma meta-cluster undergoing different stages of differentiation and maturation. The root of trajectory plasmablast showing high *XBP1* and low *SDC1* followed by short-lived plasma cells expressing high *SDC1* and long-lived plasma expressing high *SDC1*, *STAT3*, *IKZF3 and IGHA1.* The scale bar shows the level of expression.

I. Trajectory plot of macrophages depicting two distinct cell states. M1 like macrophages showing high *CD163* and *S100A12* expression and M2 like

macrophages showing high *CD163* and *FOLR2* expression. The scale bar shows the level of expression.

J. RNA *in situ* hybridisation of GC tissue sections using RNAScope 2.5 HD Duplex chromogenic Assay probed for *PLVAP* (teal) and *RGS5* (red). Cells were counterstained with hematoxylin. Analysis identifies rare double-positive (*PLVAP*/*RGS5*) cells. We observed a tendency for the *PLVAP*/*RGS5* double-positive cells to co-localize to *RGS5*-negative endothelium, however the scarcity of these double-positive cells precludes a formal assessment of the statistical significance of this association.

Supplementary Figure 3

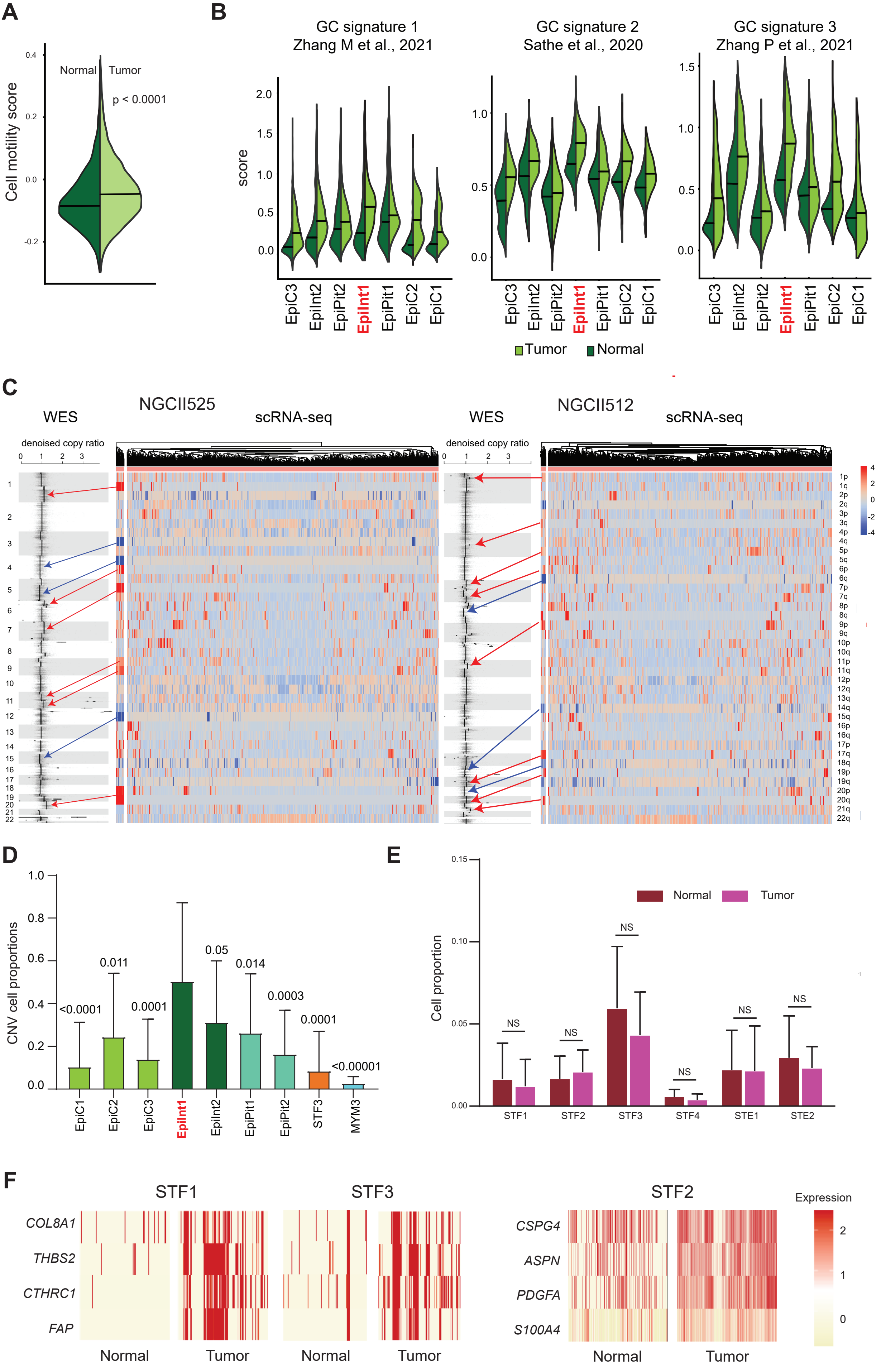

Supplementary Figure 3. Summary of tumor-normal comparisons in different lineages.

A. Split violin plot demonstrating increased expression of cell motility genes in tumor vs. normal primary GC samples.
B. Split violin plots demonstrating differences between tumor and normal samples for three GC oncogenic signatures drawn from previous single-cell studies (12-14).
C. Heatmap correlating CNVs called by scRNA-seq (using CONICSmat) and WES, demonstrating significant overlap for sample NGCII525 ($p = 0.003$) and NGCII512 ($p = 0.002$). Blue and red depicts inferred copy number loss and gain respectively.
D. Bar graph depicting proportion of cells with inferred CNVs by epithelial subcluster, with STF3 and MYM3 as negative controls. Comparisons are made between EpiInt1(labelled in red) and other subclusters (p-value above bar).
E. Bar graph depicting absolute proportions of cells in fibroblast and endothelial meta-clusters. There are no statistically significant differences between tumor and normal gastric samples.
F. Heatmap showing upregulation in tumor samples of CAF genes (*FAP, COL8A1, THBS2* and *CTHRC1*) in both STF1 and STF3 fibroblast subclusters (*LUM* associated) compared to normal. STF2 (pro-angiogenic pericytes) showed significant upregulation of different set of CAF markers (*CSPG4, ASPN, PDGFA* and *S100A4*) in tumor vs. normal comparison.

Supplementary Figure 4

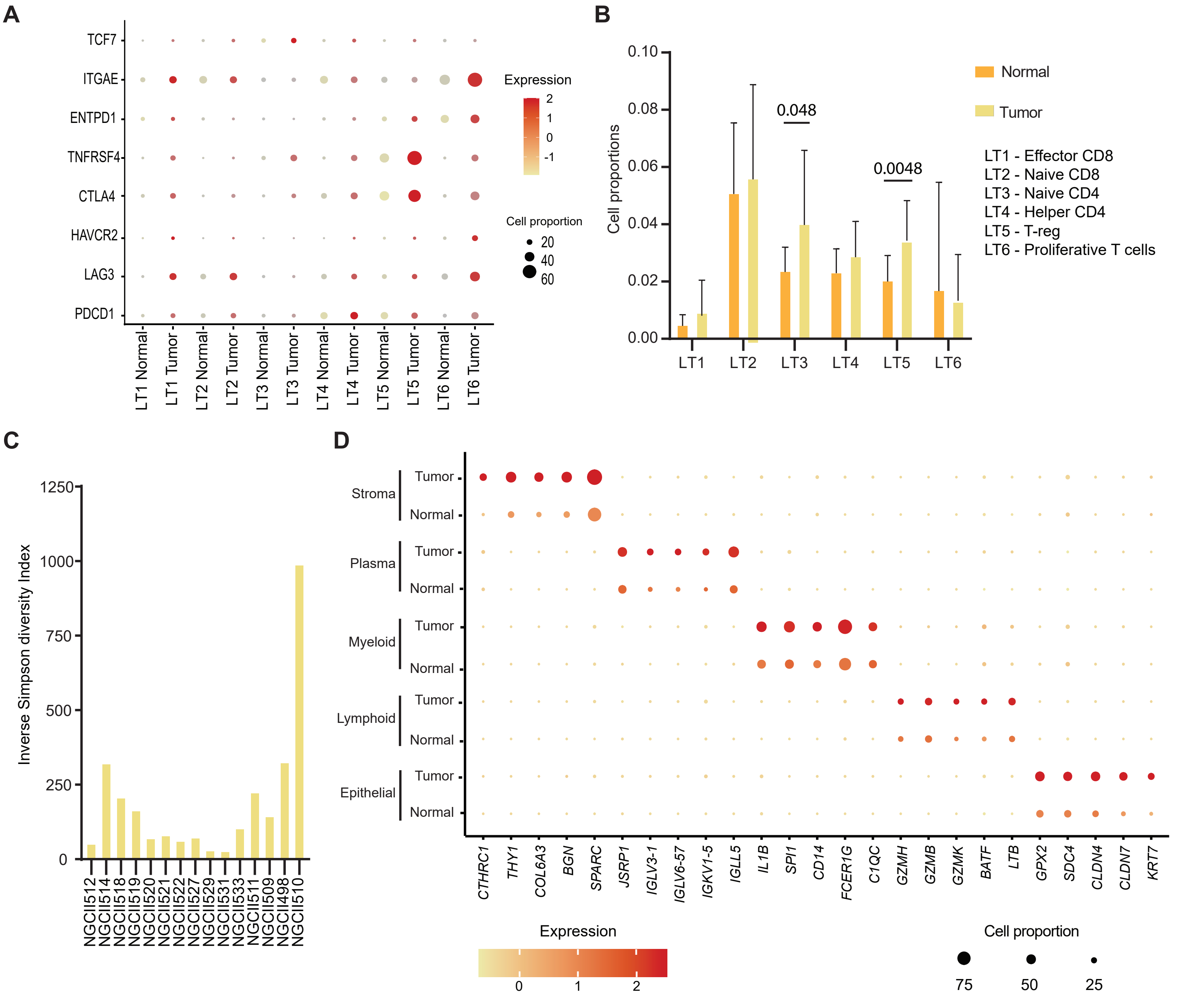

Supplementary Figure 4. scRNA-Seq T-cell analysis

A. Dot plot showing expression of immune checkpoints in T-cell subtypes of therapeutic interest in GC. *LAG3* is significantly upregulated in LT1 and LT2 (effector and naïve CD8 T-cells). *TNFRSF4* (*OX-40*) is significantly upregulated in LT3-6, but not CD8 T-cells. *CTLA4* is significantly upregulated in LT1 and LT3.
B. Bar graph depicting absolute proportions of T-cell subclusters comparing tumor vs. normal. Higher proportion of Tregs (p = 0.0048) and naïve CD4 T-cells (p = 0.048) in tumors compared to normal are observed.
C. T-cell receptor (TCR) sequencing. Assaying 19,250 cells from 15 tumor samples, 18,722 TCR sequences of full-length alpha and beta chains were obtained. On average, there were 703 unique clonotypes per sample (range: 126-2365) and of these an average of 85% were single clonotypes (average: 581 per sample). TCR diversity, measured by the Inverse Simpson index, was in the range of 34.3 to 994.
D. Bubble plot showing the expression of genes specific to the five meta-clusters in tumor and normal gastric samples. The size of the bubble represents the percentage of cells expressing these genes, while the color represents the average expression of the gene.

Supplementary Figure 5

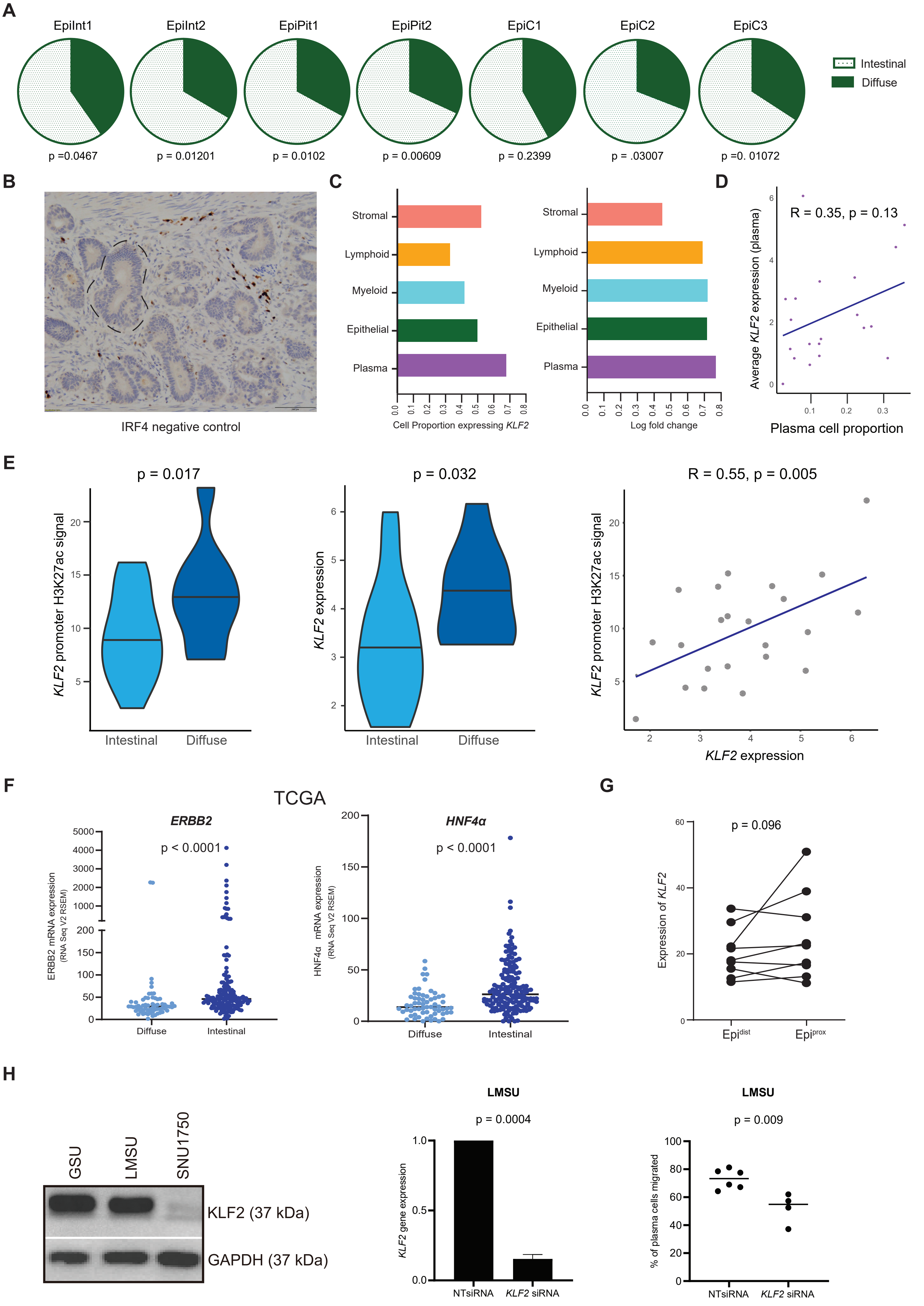

Supplementary Figure 5. Analysis of *KLF2* expression**.**

A. Pie-charts showing the relative proportions of epithelial subclusters between diffuse and intestinal subtypes. Intestinal subtype GCs are associated with an overall higher proportion of epithelial clusters, including EpiInt1 (p = 0.046).

B. Immunohistochemical staining of IRF4 showing negative staining in epithelial cells (region marked in figure).

C. Bar graph showing the proportion of cells expressing *KLF2* in different meta-clusters of diffuse GC compared to intestinal (Left). Stromal, epithelial and plasma meta-clusters had >50% of cells expressing *KLF2.* Bar graph showing the log fold change of *KLF2* in different meta-clusters of diffuse GC compared to intestinal (Right). Only the stromal meta-cluster had a log fold change <0.5.

D. Pearson correlation plot between plasma cell proportions and *KLF2*-expressing plasma cells demonstrates no statistically significant correlation.

E. Violin plot showing significantly higher *KLF2* H3K27ac promoter signal measured by ChIP-seq in diffuse GC compared to intestinal measured by (left violin); independent primary GC dataset (*n* = 24). Violin plot showing a corresponding significant increase of *KLF2* gene expression in bulk RNA-seq of the same GC samples in which ChIP-Seq was performed (right violin). Pearson correlation graph showing positive correlation between *KLF2* H3K27ac promoter signal and bulk RNA-seq expression.

F. Bee swarm plot showing within-subtype variation of genes previously associated with intestinal-type GC (*ERBB2* and *HNF4α*) in the bulk RNA-seq TCGA STAD dataset.

G. Graph showing an increased trend in the expression of *KLF2* in PanCK+ epithelial cells proximal to plasma cells (Epi^{prox}) compared to epithelial cells distal to the plasma (Epi^{dist}) in all gastric samples (n=9; paired for Epi^{prox} and Epi^{dist}). p-value computed by paired t-test.

H. Western blot (left panel) showing basal levels of KLF2 in GC cell lines GSU, LMSU and SNU1750. Transient knockdown of *KLF2* in GC cell line, LMSU results in significantly lower *KLF2* mRNA expression compared to NT [non-targeting] controls (N = 3) (middle panel). Loss of *KLF2* in LMSU significantly reduces migration of plasma cells derived from peripheral blood mononuclear cells [PBMCs] (N = 4-6) (right panel).

Supplementary Figure 6

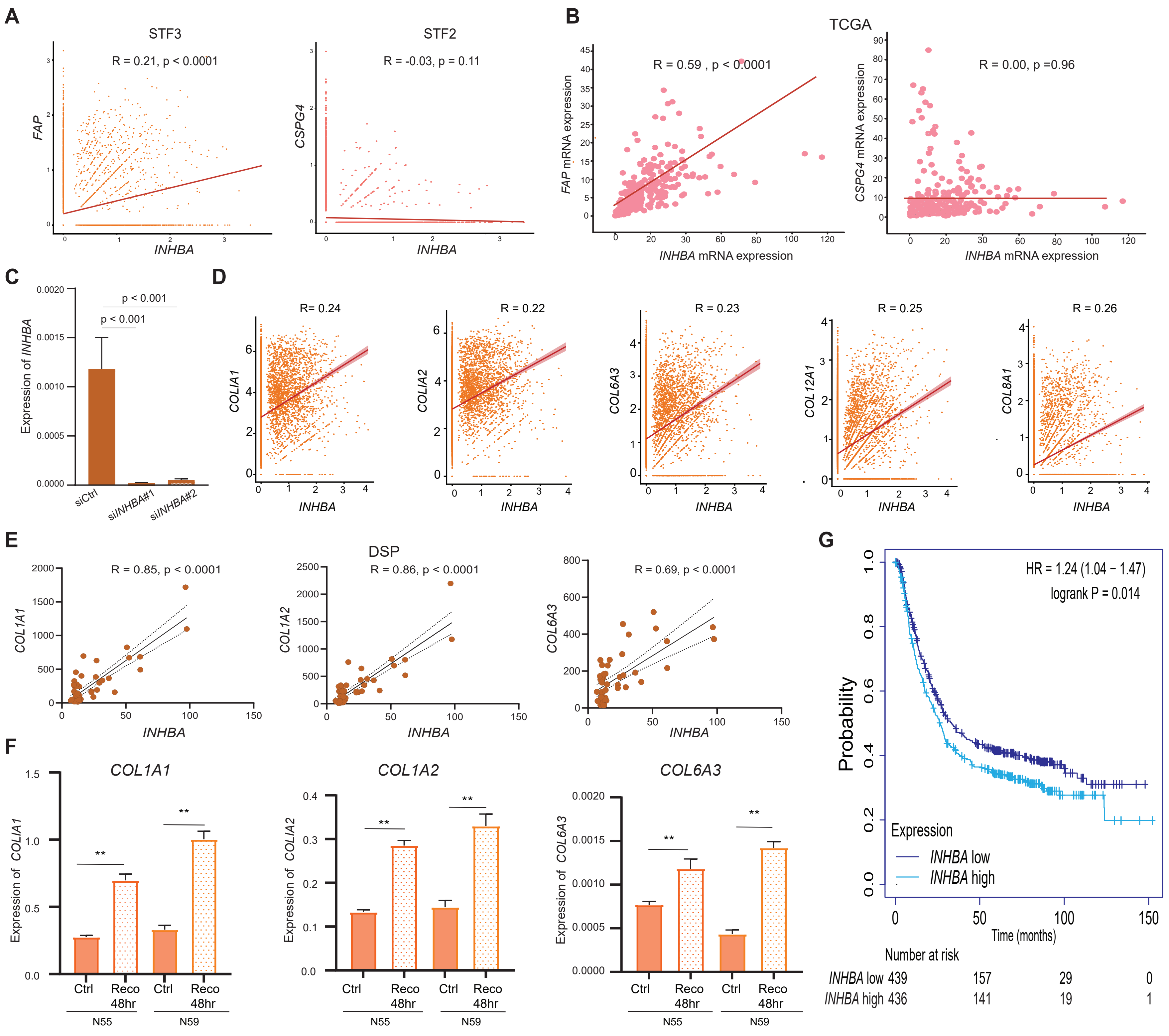

Supplementary Figure 6. Analysis of *INHBA* and *FAP* expression.

A. Left graph showing positive correlation between *INHBA* and *FAP* in STF3 (*FAP* high) fibroblast subcluster. Right graph showing no correlation between *CSPG4* and *INHBA* in STF2 (*CSPG4* high) fibroblast subcluster. p-value computed by Pearson's R.

B. Left graph showing positive correlation between *FAP* and *INHBA* in bulk RNA-seq TCGA STAD data. Right graph showing no correlation between *CSPG4* and *INHBA* in bulk RNA-seq TCGA STAD data. p-value computed by Pearson's R.

C. Knockdown efficiency and specificity of si*INHBA* 1 and si*INHBA* 2 siRNAs. *INHBA* knockdown greater than 90% was observed in CAF61 lines after 48 hrs as shown by the selective reduction of the *INHBA* transcript level after siRNA treatment as compared to control.

D. Correlation between *INHBA* and *TGF-β*-induced collagen target genes (*COL1A1, COL1A2, COL6A3, COL8A1* and *COL12A1*) in scRNA-seq CAFs

E. Correlation between *INHBA* and *TGF-β* induced collagen target genes (*COL1A1, COL1A2, COL6A3*) in DSP fibroblast ROIs.

F. Bar graphs showing the expression of collagen genes in CAFs treated with recombinant *INHBA* (Reco) for 48 hours. Significant increases in collagen genes (*COL1A1, COL1A2* and *COL6A3*) were observed in two CAF lines (N55 and N59). ** = $p < 0.01$.

G. Kaplan–Meier survival curves of pooled GC microarray data showing significant differences in patient survival between *INHBA* high and low samples.

Supplementary Figure 7

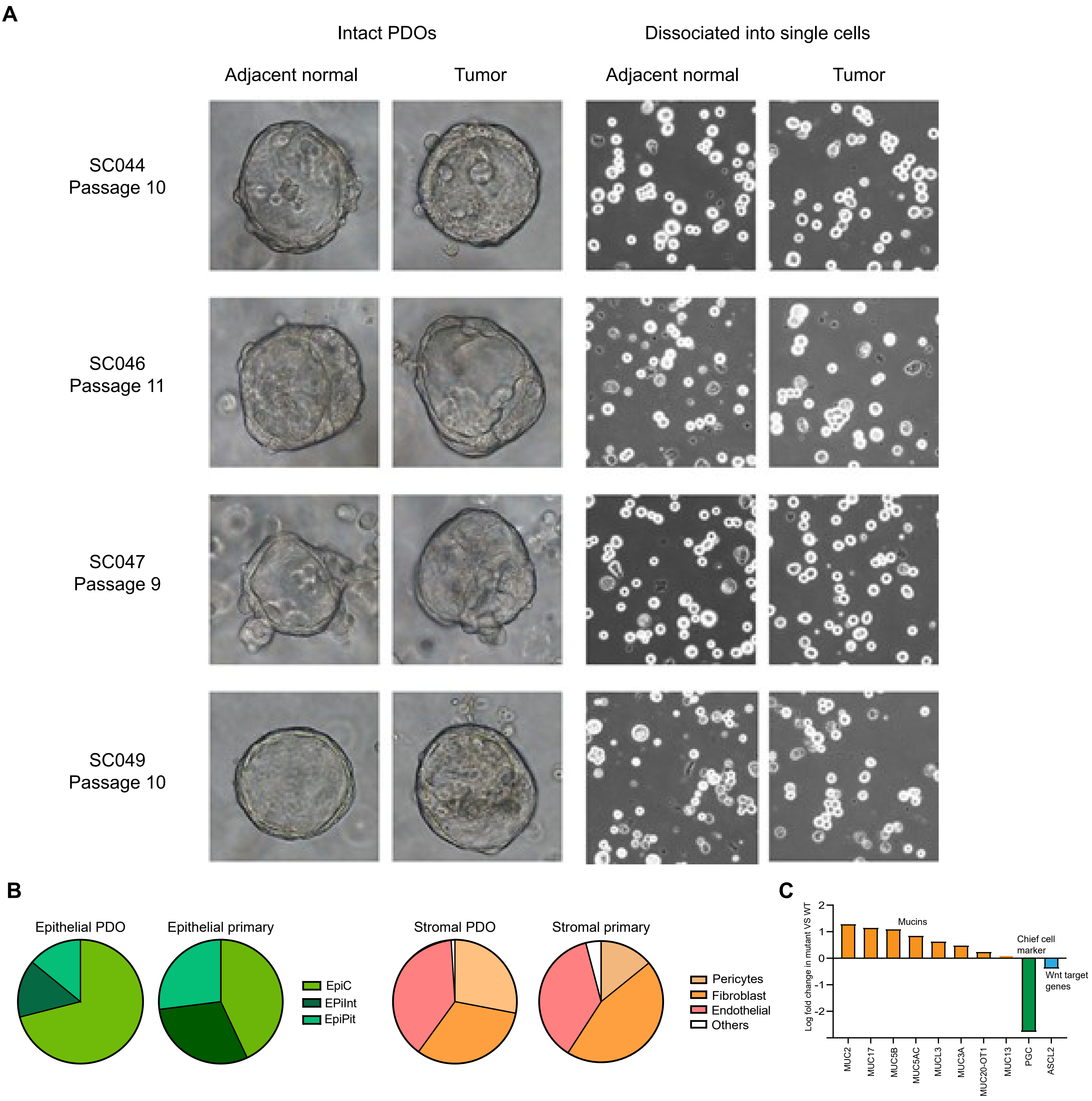

Supplementary Figure 7. PDO cultures *in vitro*.

A. Representative brightfield images of the four different cancer with matched normal PDO lines. Right columns representative images showing dissociated single cells of each sample

B. Proportions of epithelial and stromal subclusters compared between PDO and primary tumor.

C. scRNA-Seq data comparison of *ARID1A* mutated (n=2) *vs.* wildtype (WT) (n=8) in epithelial cells, showing significant upregulation of mucins (e.g. *MUC2*, *MUC5AC*) in *ARID1A* mutant samples compared to WT, along with downregulation of chief-cell markers and Wnt-target genes.
